# CmWRKY6–1–CmWRKY15-like transcriptional cascade negatively regulates the resistance to *fusarium oxysporum* infection in *Chrysanthemum morifolium*

**DOI:** 10.1093/hr/uhad101

**Published:** 2023-05-10

**Authors:** Weihao Miao, Xiangyu Xiao, Yuean Wang, Lijiao Ge, Yanrong Yang, Ye Liu, Yuan Liao, Zhiyong Guan, Sumei Chen, Weimin Fang, Fadi Chen, Shuang Zhao

**Affiliations:** College of Horticulture, Nanjing Agricultural University, Nanjing 210095, China; Key Laboratory of Landscaping, Ministry of Agriculture and Rural Affairs, Nanjing 210095, China; Zhongshan Biological Breeding Laboratory, No.50 Zhongling Street, Nanjing, Jiangsu 210014, China; College of Horticulture, Nanjing Agricultural University, Nanjing 210095, China; Key Laboratory of Landscaping, Ministry of Agriculture and Rural Affairs, Nanjing 210095, China; Zhongshan Biological Breeding Laboratory, No.50 Zhongling Street, Nanjing, Jiangsu 210014, China; College of Horticulture, Nanjing Agricultural University, Nanjing 210095, China; Key Laboratory of Landscaping, Ministry of Agriculture and Rural Affairs, Nanjing 210095, China; Zhongshan Biological Breeding Laboratory, No.50 Zhongling Street, Nanjing, Jiangsu 210014, China; College of Horticulture, Nanjing Agricultural University, Nanjing 210095, China; Key Laboratory of Landscaping, Ministry of Agriculture and Rural Affairs, Nanjing 210095, China; Zhongshan Biological Breeding Laboratory, No.50 Zhongling Street, Nanjing, Jiangsu 210014, China; College of Horticulture, Nanjing Agricultural University, Nanjing 210095, China; Key Laboratory of Landscaping, Ministry of Agriculture and Rural Affairs, Nanjing 210095, China; Zhongshan Biological Breeding Laboratory, No.50 Zhongling Street, Nanjing, Jiangsu 210014, China; College of Horticulture, Nanjing Agricultural University, Nanjing 210095, China; Key Laboratory of Landscaping, Ministry of Agriculture and Rural Affairs, Nanjing 210095, China; Zhongshan Biological Breeding Laboratory, No.50 Zhongling Street, Nanjing, Jiangsu 210014, China; College of Horticulture, Nanjing Agricultural University, Nanjing 210095, China; Key Laboratory of Landscaping, Ministry of Agriculture and Rural Affairs, Nanjing 210095, China; Zhongshan Biological Breeding Laboratory, No.50 Zhongling Street, Nanjing, Jiangsu 210014, China; College of Horticulture, Nanjing Agricultural University, Nanjing 210095, China; Key Laboratory of Landscaping, Ministry of Agriculture and Rural Affairs, Nanjing 210095, China; Zhongshan Biological Breeding Laboratory, No.50 Zhongling Street, Nanjing, Jiangsu 210014, China; College of Horticulture, Nanjing Agricultural University, Nanjing 210095, China; Key Laboratory of Landscaping, Ministry of Agriculture and Rural Affairs, Nanjing 210095, China; Zhongshan Biological Breeding Laboratory, No.50 Zhongling Street, Nanjing, Jiangsu 210014, China; College of Horticulture, Nanjing Agricultural University, Nanjing 210095, China; Key Laboratory of Landscaping, Ministry of Agriculture and Rural Affairs, Nanjing 210095, China; Zhongshan Biological Breeding Laboratory, No.50 Zhongling Street, Nanjing, Jiangsu 210014, China; College of Horticulture, Nanjing Agricultural University, Nanjing 210095, China; Key Laboratory of Landscaping, Ministry of Agriculture and Rural Affairs, Nanjing 210095, China; Zhongshan Biological Breeding Laboratory, No.50 Zhongling Street, Nanjing, Jiangsu 210014, China; College of Horticulture, Nanjing Agricultural University, Nanjing 210095, China; Key Laboratory of Landscaping, Ministry of Agriculture and Rural Affairs, Nanjing 210095, China; Zhongshan Biological Breeding Laboratory, No.50 Zhongling Street, Nanjing, Jiangsu 210014, China

## Abstract

Chrysanthemum Fusarium wilt is a soil-borne disease that causes serious economic losses to the chrysanthemum industry. However, the molecular mechanism underlying the response of chrysanthemum WRKY to *Fusarium oxysporum* infection remains largely unknown. In this study, we isolated *CmWRKY6–1* from chrysanthemum ‘Jinba’ and identified it as a transcriptional repressor localized in the nucleus via subcellular localization and transcriptional activation assays. We found that *CmWRKY6–1* negatively regulated resistance to *F. oxysporum* and affected reactive oxygen species (ROS) and salicylic acid (SA) pathways using transgenic experiments and transcriptomic analysis. Moreover, *CmWRKY6–1* bound to the W-box element on the *CmWRKY15-like* promoter and inhibited its expression. Additionally, we observed that *CmWRKY15-like* silencing in chrysanthemum reduced its resistance to *F. oxysporum* via transgenic experiments. In conclusion, we revealed the mechanism underlying the CmWRKY6–1–CmWRKY15-like cascade response to *F. oxysporum* infection in chrysanthemum and demonstrated that *CmWRKY6–1* and *CmWRKY15-like* regulates the immune system.

## Introduction

Fusarium wilt is a common plant disease caused by *Fusarium oxysporum*. *F. oxysporum* is the main pathogen invading the roots of plants, causing root rot, vascular blockage, leaf yellowing, and wilting, eventually leading to plant death due to a lack of water supply to the plant [1–3]. Fusarium wilt causes serious economic losses to the industry, necessitating the development of new disease-resistant varieties to control this disease. Plants have developed specific innate immune mechanisms during evolution to combat such pathogen infections. One such defense mechanism is the pathogen-associated molecular pattern (PAMP)-triggered immunity (PTI), which is activated by the specific recognition of PAMPs by pattern recognition receptors on the plant cell surface [4]. However, some pathogens can evade PTI by counteracting these factors, in which case, the plants activate the second mechanism of defense, effector-triggered immunity (ETI), by secreting resistance proteins specifically recognizing the pathogen effectors [5]. ETI is more rapid and intense than PTI. A complex immune network is involved in PTI and ETI for efficient immune regulation in plants [6]. Transcription factors affect the downstream immune system by identifying and regulating target genes.

WRKY is a plant-specific transcription factor that regulates plant biotic and abiotic stress responses and plays important regulatory roles in the plant immune response [7–9]. WRKY contains a WRKYGQK sequence at its N-terminal end and a zinc finger structure at its C-terminal end. Zinc finger structure is divided into C-X_4–5_-C-X_22–23_-H-X_1_-H and C-X_7_-C-X_23_-H-X_1_-C. WRKY family is divided into three major groups: I, II, and III [10]. Group I contains two WRKYGOK sequences and one C-X_4–5_-C-X_22–23_-H-X_1_-H structure, group II contains one WRKYGQK sequence and one C-X_4–5_-C-X_22–23_-H-X_1_-H structure, and group III contains one WRKYGQK sequence and one C-X_7_-C-X_23_-H-X_1_-C structure [11, 12]. WRKY plays an important role in the plant immune response. *AtWRKY28/75* and *AtWRKY33* increase the plant resistance to *Sclerotinia sclerotiorum* and *Botrytis cinerea*, respectively [13, 14]. *OsWRKY45* and *OsWRKY53* increase the plant resistance to the rice blast pathogen, *Magnaporthe grisea* [15, 16]. In addition, WRKY can specifically recognize and bind to W-box elements (TTGACC/T). W-box elements are present in the promoters of many plant defence-related genes, and WRKY can regulate plant defence-related genes, thereby affecting the plant defense systems [17]. OscWRKY1 positively regulates the expression levels of the phenylpropanoid pathway genes by binding to W-box elements in *PAL* and *C4H* promoters, thereby enhancing the plant resistance to bacteria [18]. Phosphorylated CaWRKY64 binds to the W-box element in the *CaEDS1* promoter and enhances the resistance to *F. oxysporum* in chickpeas [19]. Soybean GmWRKY31 binds to the W-box element in the downstream *GmSAGT1* promoter, increasing *GmSAGT1* transcription and enhancing plant resistance to soybean downy mildew [20].

Reactive oxygen species (ROS), including hydrogen peroxide (H_2_O_2_), superoxide anions (O2-), and hydroxyl radicals (-OH), are produced by plants in response to adversity [21]. ROS can act as second messengers to trigger the plant defense response. However, excessive accumulation of ROS can cause serious damage to proteins and nucleic acids, eventually leading to programmed cell death in plants [22]. To maintain the balance of ROS, plants have specific enzymes, such as peroxidase (POD), catalase (CAT), polyphenol oxidase (PPO), glutathione reductase, and ascorbate peroxidase [23]. POD, CAT, and PPO can be used as physiological indices to evaluate the plant resistance to pathogens [24, 25]. WRKY is a key transcription factor regulating ROS production. WRKY8 activates *RBOHB* expression and induces a hypersensitivity response in tobacco [26]. WRKY1 activates long non-coding RNA (lncRNA)-33 732 to induce *RBOH* expression, promote H_2_O_2_ production, and improve tomato resistance to *Phytophthora infestans* [27].

Salicylic acid (SA) has been suggested to be an ROS homeostasis regulator in plants. SA is a phenolic compound with antioxidant properties that plays an important regulatory role in the immune response of plants [28, 29]. SA increases the levels of glutathione, inducing the elimination of ROS in plants [30, 31]. In addition, WRKY can participate in plant immunity by regulating the SA pathway. Inhibition of SA signaling by *CaWRKY70* increases the susceptibility of chickpeas to *F. oxysporum* [32]*.* We previously reported that *CmWRKY8–1* affects resistance to *F. oxysporum* by regulating the genes involved in the SA synthesis pathway in chrysanthemum [33].

Chrysanthemum is extensively cultivated in China and loved by the general population. However, Fusarium wilt causes serious damage to chrysanthemum plants. At present, there are more and more studies on the mining of chrysanthemum resistance functional genes [34, 35]. Identifying the molecular mechanisms underlying the chrysanthemum response to *F. oxysporum* infection is necessary to manage this pathogen. Song reported that *CmWRKY6* is involved in *F. oxysporum* infection, suggesting its involvement in the response of chrysanthemum to *F. oxysporum* infection [36]. In this study, we cloned *CmWRKY6–1* and found that it negatively regulated resistance to *F. oxysporum* and affected ROS and SA pathways. We also identified a regulatory relationship between *CmWRKY6–1* and *CmWRKY15-like*. Overall, we revealed a novel pathway for chrysanthemum response to *F. oxysporum* infection that may be used as a basis for future disease resistance studies of chrysanthemum WRKYs.

## Results

### Sequence analysis of CmWRKY6–1

**Figure 1:**
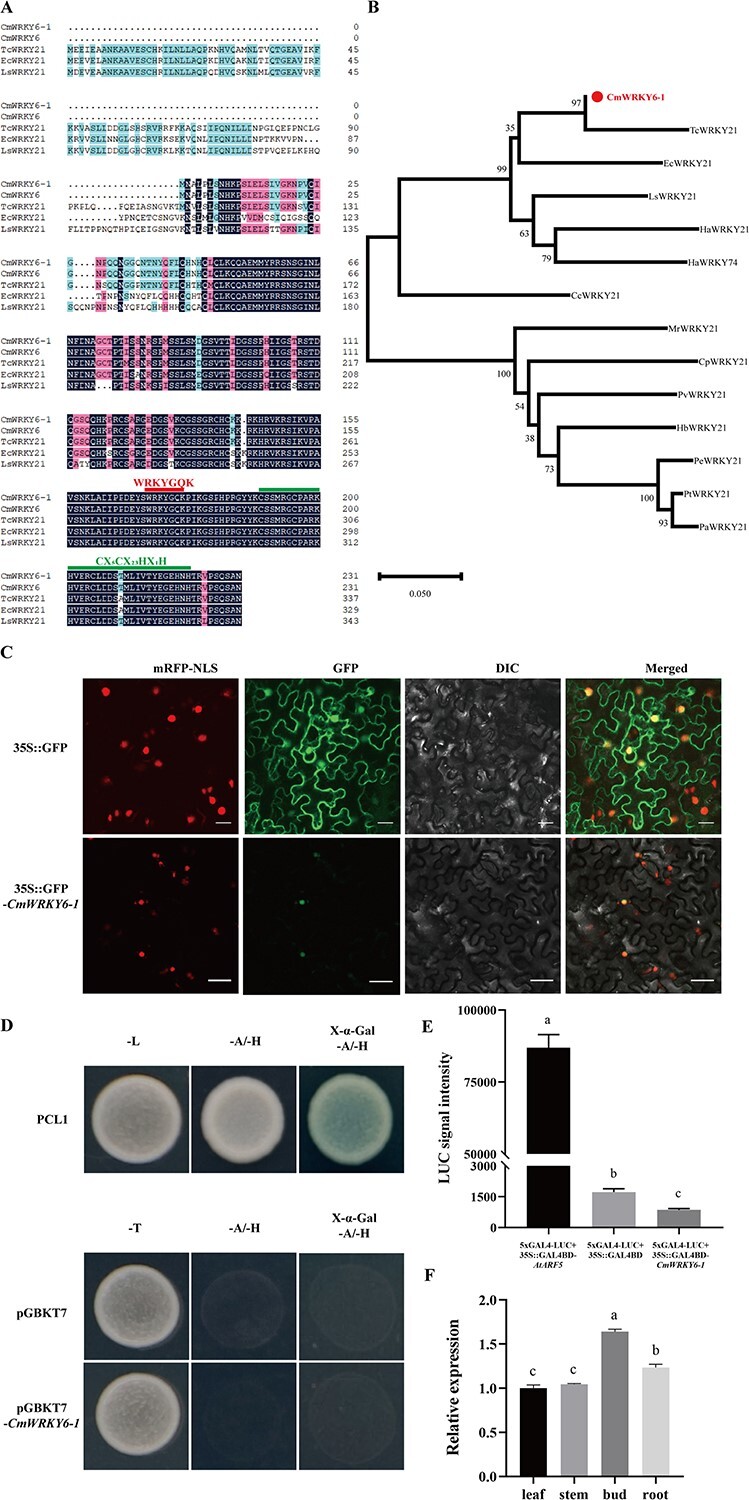
Sequence analysis and characteristics of CmWRKY6–1. **A** Comparisons of CmWRKY6–1 sequence with other homologous sequences of different species. WRKYGQK domain is indicated by the red line, and the zinc finger domain is indicated by the green line. **B** Phylogenetic analysis of CmWRKY6–1 (marked in red). Remaining sequences contain *Tanacetum cinerariifolium* TcWRKY21 (GenBank accession no. GEW55685.1), *Erigeron canadensis* EcWRKY21 (GenBank accession no. XP_043635710.1), *Lactuca sativa* LsWRKY21 (GenBank accession no. XP_023733377.1), *Helianthus annuus* HaWRKY21 (GenBank accession no. XP_021987406.1), *H. annuus* HaWRKY74 (GenBank accession no. XP_035840018.1), *Cynara cardunculus* CcWRKY21 (GenBank accession no. XP_024968179.1), *Morella rubra* MrWRKY21 (GenBank accession no. KAB1208866.1), *Carica papaya* CpWRKY21 (GenBank accession no. XP_021898029.1), *Pistacia vera* PvWRKY21 (GenBank accession no. XP_031255336.1), *Hevea brasiliensis* HbWRKY21 (GenBank accession no. XP_021672672.1), *Populus euphratica* PeWRKY21 (GenBank accession no. XP_011028741.1), *Populus trichocarpa* PtWRKY21 (GenBank accession no. XP_002302070.2), and *Populus alba* PaWRKY21 (GenBank accession no. XP_034915120.1). **C** Subcellular localization of CmWRKY6–1. 35S::GFP for pORE-R4 and 35S::GFP-*CmWRKY6–1* for pORE-R4-*CmWRKY6–1*. mRFP-NLS: images taken in the red fluorescence channel; GFP: images taken in the green fluorescence channel; DIC: images taken in the bright light channel; Merged: overlay plots. Bar = 20 μm. **D** Validation of the transcriptional activation of CmWRKY6–1 by the yeast system. **E** Validation of the transcriptional activation of CmWRKY6–1 by chrysanthemum protoplasts. Different letters indicate significant differences according to Duncan’s multiple range test; *P* < 0.05; same scheme applies below. **F** Expression levels of *CmWRKY6–1* in different tissues of chrysanthemum during the nutritional growth period.

In previous experiments, Song found that *CmWRKY6* (KC615360) responds to *F. oxysporum* infection [36]. To identify the mechanism by which *CmWRKY6* responds to *F. oxysporum* infection, we cloned and isolated a gene sequence with a 699 bp open reading frame (ORF) from ‘Jinba’ variety (Table S1, see online supplementary material). Homologous sequence analysis revealed that the protein sequence contained a WRKY structural domain with the zinc finger structure, C-X_5_-C-X_23_-H-X_1_-H, belonging to WRKY family II. Notably, it has only one amino acid difference from the CmWRKY6 protein sequence; therefore, we named it CmWRKY6–1 (Fig. 1A). Phylogenetic tree revealed that CmWRKY6–1 has the highest affinity for *Tanacetum cinerariifolium* TcWRKY21 (Fig. 1B).

### Characteristics of CmWRKY6–1

We transiently transformed tobacco plants with the pORE-R4-*CmWRKY6–1* and found that CmWRKY6–1 was localized in the nucleus (Fig. 1C).

Next, we transformed pGBKT7-*CmWRKY6–1* in a yeast two-hybrid (Y2H). We found that transformed with the negative controls pGBKT7 and pGBKT7-*CmWRKY6–1* did not grow normally on SD/Ade/His-deficient medium (Fig. 1D). Furthermore, we used chrysanthemum protoplasts to conduct transcriptional activity analysis. Luciferase (LUC) fluorescence signal intensity of CmWRKY6–1 was significantly lower than that of the positive control (Fig. 1E). These results indicate that CmWRKY6–1 does not exhibit any transcriptional activity.

To clarity the expression patterns of *CmWRKY6–1* in different chrysanthemum tissues during growth, the terminal buds, stems, leaves, and roots of ‘Jinba’ were sampled and analysed via qRT-PCR. Relative expression levels of *CmWRKY6–1* were the highest in buds, followed by the roots (Fig. 1F).

### *CmWRKY6–1* negatively regulates the resistance of ‘Jinba’ to *F. oxysporum*

**Figure 2:**
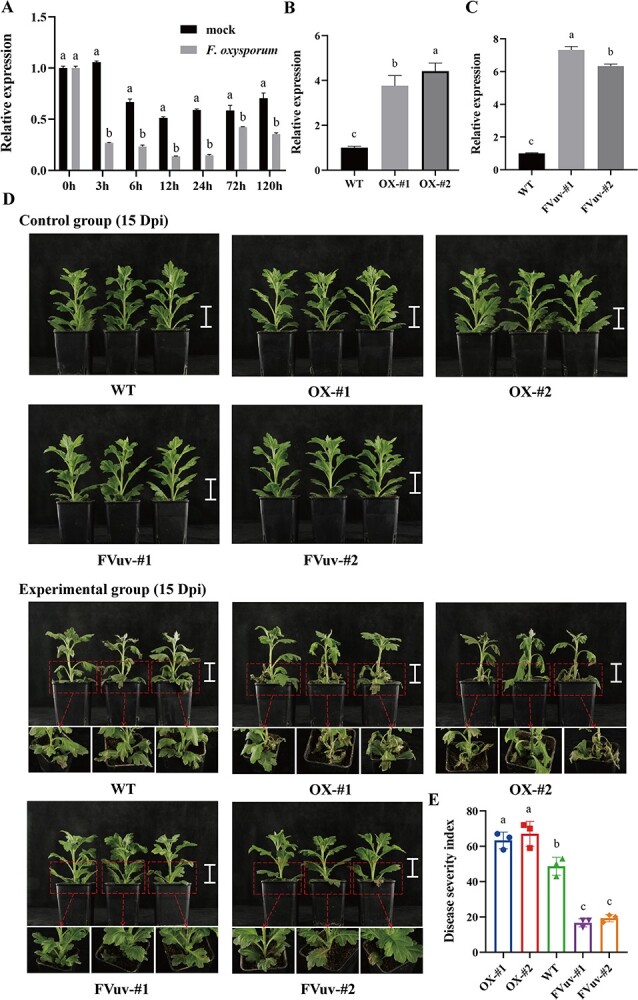
Pathogenesis phenotypes of *CmWRKY6–1* transgenic lines. **A** Expression levels of *CmWRKY6–1* in response to *Fusarium oxysporum* infection in ‘Jinba’ variety. **B***CmWRKY6–1* relative expression levels in *CWRKY6–1* overexpression (*CWRKY6–1*-OX) and wild-type (WT) lines. **C***CmWRKY6–1* relative expression levels in FVuv-*CmWRKY6–1* and WT lines. **D** Phenotypic observations of *CmWRKY6–1* transgenic and WT plants after inoculation with *F. oxysporum*. Control group inoculated with potato dextrose broth (PDB); experimental group inoculated with *F. oxysporum*. Bar = 5 cm. **E** Disease severity index (DSI) of each line.

To investigate the expression patterns of *CmWRKY6–1* in response to *F. oxysporum* infection, we performed qRT-PCR assays on the roots of chrysanthemum ‘Jinba’ varieties after inoculation with *F. oxysporum*. Expression levels of *CmWRKY6–1* were significantly more downregulated in the experimental group than in the control group at 3 h. Moreover, expression levels of *CmWRKY6–1* were significantly lower in the experimental group than in the control group within 120 h (Fig. 2A).

To further determine the functions of *CmWRKY6–1* in chrysanthemum, we transfected the pORE-R4-*CmWRKY6–1* plasmid into ‘Jinba’ and obtained two *CmWRKY6–1* overexpression lines, OX-#1 and OX-#2 (Fig. S1, see online supplementary material). qRT-PCR revealed that the expression levels of *CmWRKY6–1* in the overexpression lines were 3.7- and 4.4-fold higher than those in the wild-type (WT), respectively (Fig. 2B). As CmWRKY6–1 did not exhibit any transcriptional activation activity, we constructed the FVuv-R4-*CmWRKY6–1* vector by fusing CmWRKY6–1 with VP64 to convert CmWRKY6–1 into a transcriptional activator [33]. The vector was then transferred into ‘Jinba’ to obtain two *CmWRKY6–1* interfering lines, Fvuv-#1 and Fvuv-#2 (Fig. S2, see online supplementary material). Expression levels of *CmWRKY6–1* were 7.3- and 6.3-fold higher in the interfering lines than in the WT, respectively (Fig. 2C). Next, we simultaneously inoculated the WT and *CmWRKY6–1* transgenic lines with *F. oxysporum*. Basal leaf wilting was more severe in OX lines than in WT and Fvuv lines (Fig. 2D; Fig. S3, see online supplementary material). In contrast, the basal leaves of Fvuv lines exhibited only a slightly wilted phenotype and were significantly less affected than WT and OX lines (Fig. 2D; Fig. S3, see online supplementary material). We also calculated the disease severity index (DSI) of each line and found that OX lines had higher DSI than WT, whereas Fvuv lines had lower DSI than WT (Fig. 2E). These results suggest that *CmWRKY6–1* negatively regulates resistance to *F. oxysporum* in chrysanthemum.

### Transcriptome analysis of *CmWRKY6–1* overexpression line

To further determine the mechanism underlying the *CmWRKY6–1* response to *F. oxysporum* infection, we conducted transcriptome analyses of root samples from OX-#1 and WT lines 0, 3, and 72 h after inoculation (control group: WT-0 h, WT-3 h, and WT-72 h; experimental group: OX-0 h, OX-3 h, and OX-72 h). Correlation coefficients revealed high repeatability of samples within each group and differences among groups (Fig. S4, see online supplementary material), and the percentage of Q30 bases in all samples was ≥90% (Table S2, see online supplementary material). Next, we explored the differentially expressed genes (DEGs) between the control and experimental groups at each time point and identified 9229, 3587, and 3376 DEGs at 0, 3, and 72 h, respectively (Fig. S5, see online supplementary material).

### *CmWRKY6–1* negatively regulates the ability to eliminate ROS in chrysanthemum

**Figure 3:**
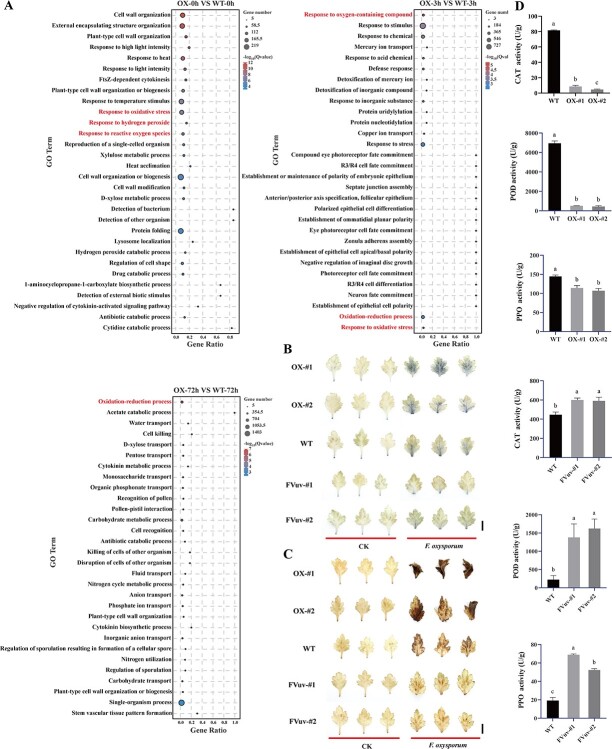
*CmWRKY6–1* responds to *F. oxysporum* infection by regulating the reactive oxygen species (ROS) pathway. **A** Gene Ontology (GO) enrichment analysis of the DEGs. GO terms associated with redox pathways are marked in red. **B** Nitrotetrazolium blue chloride (NBT) staining of transgenic and WT lines. Bar = 1 cm. **C** Diaminobenzidine (DAB) staining of transgenic and WT lines. Bar = 1 cm. **D** Determination of the activities of enzymes related to the redox pathway.

We further conducted Gene Ontology (GO) analysis of DEGs at various time points and found that redox processes, including responses to ROS, were significantly enriched (Fig. 3A). We hypothesized that *CmWRKY6–1* regulates the redox pathway.

To verify this hypothesis, we inoculated transgenic and WT lines with *F. oxysporum* and stained the infected basal leaves with nitrotetrazolium blue chloride (NBT) and diaminobenzidine (DAB). Levels of ROS were higher in the leaves of the inoculated *CmWRKY6–1*-OX lines than in those of the WT and FVuv-*CmWRKY6–1* lines, whereas the opposite trend was observed in the FVuv-*CmWRKY6–1* lines (Fig. 3B and C). We also measured the activities of CAT, POD, and PPO-related redox pathway enzymes in each line. *CmWRKY6–1*-OX lines had lower levels of related enzyme activities than the WT, whereas the opposite trend was observed in the FVuv-*CmWRKY6–1* lines (Fig. 3D). These results indicate that *CmWRKY6–1* negatively regulates the ability to eliminate ROS in chrysanthemum.

### *CmWRKY6–1* negatively regulates the synthesis of endogenous SA in chrysanthemum

**Figure 4:**
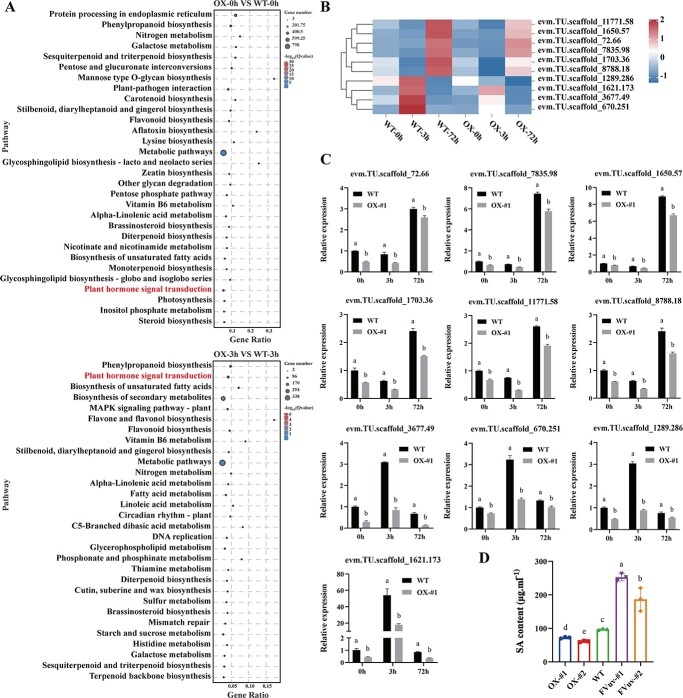
*CmWRKY6–1* responds to *F. oxysporum* infection by regulating the salicylic acid (SA) pathway. **A** Kyoto Encyclopedia of Genes and Genomes (KEGG) enrichment analysis of DEGs. Plant hormone signal transduction pathway is marked in red. **B** Expression levels of SA pathway-related genes in the transcriptome. **C** Relative quantification of the expression levels of SA pathway-related genes via quantitative reverse transcription-polymerase chain reaction (qRT-PCR). **D** Endogenous SA levels.

Kyoto Encyclopedia of Genes and Genomes (KEGG) enrichment analysis of DEGs revealed a significant enrichment in the plant hormone signal transduction pathway (Fig. 4A). We previously reported that chrysanthemum WRKY responds to *F. oxysporum* infection by regulating the SA pathway [33]. To determine whether *CmWRKY6–1* is involved in the SA pathway, we further analysed the changes in the expression levels of key genes related to the SA pathway using the transcriptome data after plant inoculation. Levels of *PAL* (evm.TU.scaffold_72.66/7835.98/1650.57), *PR2* (evm.TU.scaffold_1703.36/11771.58/8788.18), and *PR5* (evm.TU.scaffold_3677.49/670.251/1289.286/1621.173) were significantly more decreased in *CmWRKY6–1*-OX lines than in the WT (Fig. 4B). To validate the RNA-seq results, we measured the expression levels of these genes via qRT-PCR. qRT-PCR results were consistent with those of RNA-seq (Fig. 4C). Furthermore, we determined the levels of endogenous SA after *F. oxysporum* inoculation. Indeed, *CmWRKY6–1*-OX lines had lower endogenous SA levels than the WT, whereas the opposite trend was observed in the FVuv-*CmWRKY6–1* lines (Fig. 4D). In conclusion, we demonstrate that *CmWRKY6–1* negatively regulates the synthesis of endogenous SA in chrysanthemum.

### *CmWRKY6–1* inhibits the expression of *CmWRKY15-like*

In a previous report, *CmWRKY15–1* positively regulates chrysanthemum resistance to *Puccinia horiana* Henn via the ROS and SA pathways [37, 38]. In the above experiments, we also found that *CmWRKY6–1* can regulate the ROS and SA pathways. We hypothesized that there is a direct or indirect relationship between *CmWRKY6–1* and *CmWRKY15–1* in the ROS and SA pathways to regulate downstream defense systems.

**Figure 5:**
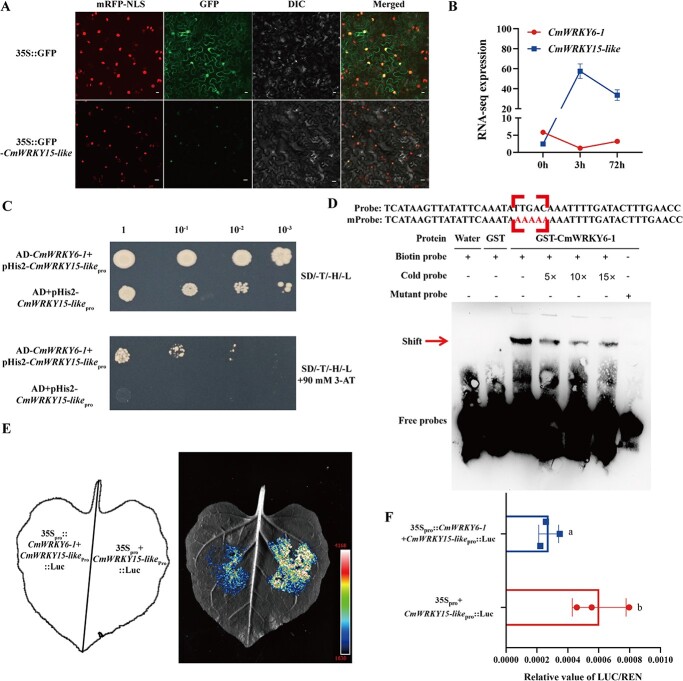
*CmWRKY6–1* inhibits the expression of *CmWRKY15-like*. **A** CmWRKY15-like subcellular localization. 35S::GFP for pORE-R4 and 35S::GFP-*CmWRKY15-like* for pORE-R4-*CmWRKY15-like*. mRFP-NLS: images taken in the red fluorescence channel; GFP: images taken in the green fluorescence channel; DIC: images taken in the bright light channel; Merged: overlay plots. Bar = 20 μm. **B** Expression levels of *CmWRKY6–1* and *CmWRKY15-like* in the transcriptome. **C** Validation of CmWRKY6–1 binding to the *CmWRKY15-like* promoter using yeast one-hybrid assay. AD for pGADT7 and pGADT7-*CmWRKY6–1* for AD-*CmWRKY6–1*. The yeast concentration was diluted 10-fold sequentially from left to right. **D** Validation of CmWRKY6–1 binding to the W-box element in the *CmWRKY15-like* promoter via electrophoretic mobility shift assay (EMSA). Red box indicates the location of the mutation; mProbe indicates the mutant probe. From left to right: biotin-labeled probe and water; GST protein and biotin-labeled probe; GST-CmWRKY6–1 protein and biotin-labeled probe; GST-CmWRKY6–1 protein and biotin-labeled probe with 5 × unlabeled probe; GST-CmWRKY6–1 protein and biotin-labeled probe with 10 × unlabeled probe; GST-CmWRKY6–1 protein and biotin-labeled robe with 15 × unlabeled probe; and GST-CmWRKY6–1 protein and mutant probe. **E** Luciferase assay of tobacco leaves. 35S_pro_ for pORE-R4, 35S_pro_::*CmWRKY6–1* for pORE-R4-*CmWRKY6–1*, and *CmWRKY15-like*_pro_::Luc for pGreenII 0800-Luc-*CmWRKY15-like*_pro_. **F** Chrysanthemum protoplast luciferase assay. 35S_pro_ for pORE-R4, 35S_pro_::*CmWRKY6–1* for pORE-R4-*CmWRKY6–1*, and *CmWRKY15-like*_pro_::Luc for pGreenII 0800-Luc-*CmWRKY15-like*_pro_.

To verify this hypothesis, we designed primers (Table S3, see online supplementary material) and cloned an ORF of 807 bp (Table S1, see online supplementary material). We then compared the obtained sequence with the CmWRKY15 sequence and identified only three amino acid differences between the two, accounting for 97% similarity of the obtained sequence to CmWRKY15–1 sequence (Fig. S6, see online supplementary material). Moreover, the obtained sequence was localized in the nucleus (Fig. 5A), and hence, named *CmWRKY15-like*. Notably, we found that the expression levels of *CmWRKY6–1* and *CmWRKY15-like* were negatively correlated using transcriptomic data (Fig. 5B). The relative expression of *CmWRKY15-like* was lower in CmWRKY6–1-OX lines than in CmWRKY6–1-FVuv lines (Fig. S7, see online supplementary material). We also cloned the promoter fragment of *CmWRKY15-like* with a length of 2048 bp (Table S4, see online supplementary material). Using the yeast single-hybrid assay and EMSA, we verified that CmWRKY6–1 bound to the W-box element in the *CmWRKY15-like* promoter (Fig. 5C and D). In addition, CmWRKY6–1 inhibited *CmWRKY15-like* expression in the dual-luciferase system (Fig. 5E and F). These results indicate that CmWRKY6–1 directly inhibits *CmWRKY15-like* expression.

### *CmWRKY15-like* silencing negatively regulates ‘Jinba’ resistance to *F. oxysporum*

**Figure 6:**
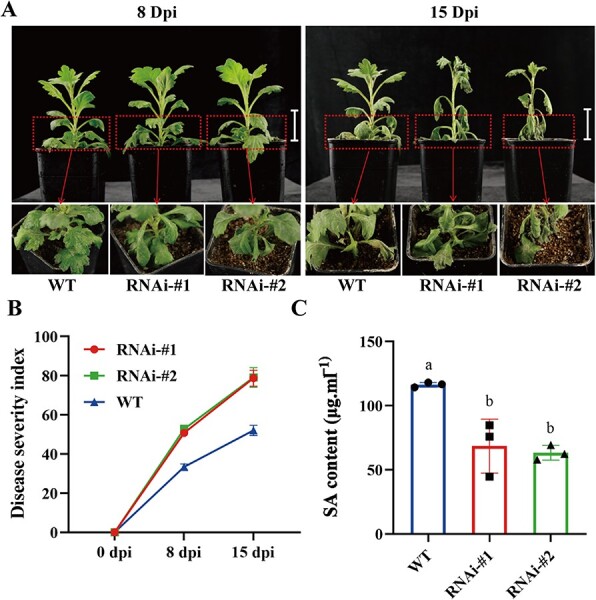
Determination of *CmWRKY15-like* functions. **A** Phenotypic changes in *CmWRKY15-like*-silenced lines after inoculation with *F. oxysporum*. Left: phenotype on the eighth day of onset; right: phenotype on the fifteenth day of onset. **B** DSI of each line. **C** Endogenous SA levels.

After determining that CmWRKY6–1 directly inhibited the downstream *CmWRKY15-like* expression, we investigated the specific functions of *CmWRKY15-like* in response to *F. oxysporum* infection. We obtained *CmWRKY15-like* interfering lines (RNAi-#1 and RNAi-#2; Fig. S8, see online supplementary material) and inoculated them with *F. oxysporum* to observe the resultant phenotype. *CmWRKY15-like* interfering lines exhibited a higher degree of pathogenic phenotype than the WT (Fig. 6A). We also calculated DSI of each line and found that RNAi lines had higher DSI than WT (Fig. 6B). In addition, endogenous SA levels of the interfering lines were lower than those of the WT (Fig. 6C). These results indicate that *CmWRKY15-like* silencing negatively regulates chrysanthemum resistance to *F. oxysporum*.

## Discussion

### *CmWRKY6–1* negatively regulates chrysanthemum resistance to *F. oxysporum* and affected ROS and SA pathways

WRKY is an important transcription factor for plant resistance that regulates defense responses against pathogens in various plants, such as rice, apple, and grapevine plants [39–41]. In our previous study on chrysanthemum Fusarium wilt, we reported that *CmWRKY8–1* affects resistance to *F. oxysporum* by regulating SA-related synthetic genes, such as *ICS1*, *PAL*, *PR1*, *PR2*, and *PR5* [33]. In this study, we identified *CmWRKY6–1* as a nuclear transcriptional repressor. Through transgene functional validation, we confirmed that *CmWRKY6–1* negatively regulated chrysanthemum resistance to *F. oxysporum*.

Comparison of the *CmWRKY6–1* overexpression and WT lines transcriptome data revealed that DEGs in the ROS and SA pathways were significantly enriched. Although the PTI and ETI responses belong to two levels of defense, they can jointly regulate ROS and SA signaling pathways to induce downstream defense responses [42]. ROS are metabolites of oxygen and its derivatives. Plants can rapidly produce ROS under abiotic and biotic stresses, which can increase their susceptibility to necrotrophic pathogens as well as their resistance to live trophic pathogens [43]. With the increase in stress degree and time, the ability of plants to eliminate ROS is seriously affected, and the basic metabolic pathways of plants are damaged [44]. SA is an important plant defense hormone that has been reported in *Arabidopsis thaliana* and tobacco [45]. SA was reported to respond to *F. oxysporum* infection in a previous study on chrysanthemum Fusarium wilt [46]. We confirmed that *CmWRKY6–1* negatively regulated ROS scavenging using NBT and DAB staining as well as enzyme activity assays. We further demonstrated that *CmWRKY6–1* inhibited SA synthesis by reducing the expression levels of SA pathway-related genes. In conclusion, *CmWRKY6–1* inhibited ROS elimination and endogenous SA synthesis.

### *CmWRKY6–1* inhibits the expression of *CmWRKY15-like*

WRKY family regulates gene expression by recognizing and binding to W-box elements [47]. Chrysanthemum CmWRKY15–1 binds to the W-box element in *CmNPR1* and interacts with CmNPR1 to improve its resistance to white rust [38]. In addition, WRKYs have been suggested to have mutual regulatory mechanism. When infected with *F. oxysporum*, chickpea CaWRKY40 binds to the W-box element in the *CaWRKY33* promoter, enhancing defense against pathogens [48]. Pepper *CaWRKY40* is both self-regulated and regulated by other WRKYs [49]. We have noticed that both CmWRKY15–1 and CmWRKY6–1 can regulate the ROS and SA pathways in chrysanthemum [37, 38]. So, we hypothesized that there is a direct or indirect relationship between *CmWRKY6–1* and *CmWRKY15–1* in the ROS and SA pathways.

We undertook the experiment to support the hypothesis. In this study, we obtained an additional copy of *CmWRKY15–1*, *CmWRKY15-like*, that exhibited 97% similarity with it. CmWRKY6–1 inhibited the expression of *CmWRKY15-like* by binding to the W-box element in the *CmWRKY15-like* promoter. When *CmWRKY15-like* was silenced, chrysanthemum plant was susceptible to *F. oxysporum*, indicating that *CmWRKY15-like* is functionally important for chrysanthemum to resist *F. oxysporum* infection. Our results revealed the regulatory relationship between CmWKRKY6–1 and CmWRKY15-like, providing a new theoretical basis for the disease resistance regulatory network among WRKYs.

### Potential action mechanism of *CmWRKY6–1–CmWRKY15-like* cascade in inhibiting plant resistance to *F. oxysporum*

**Figure 7:**
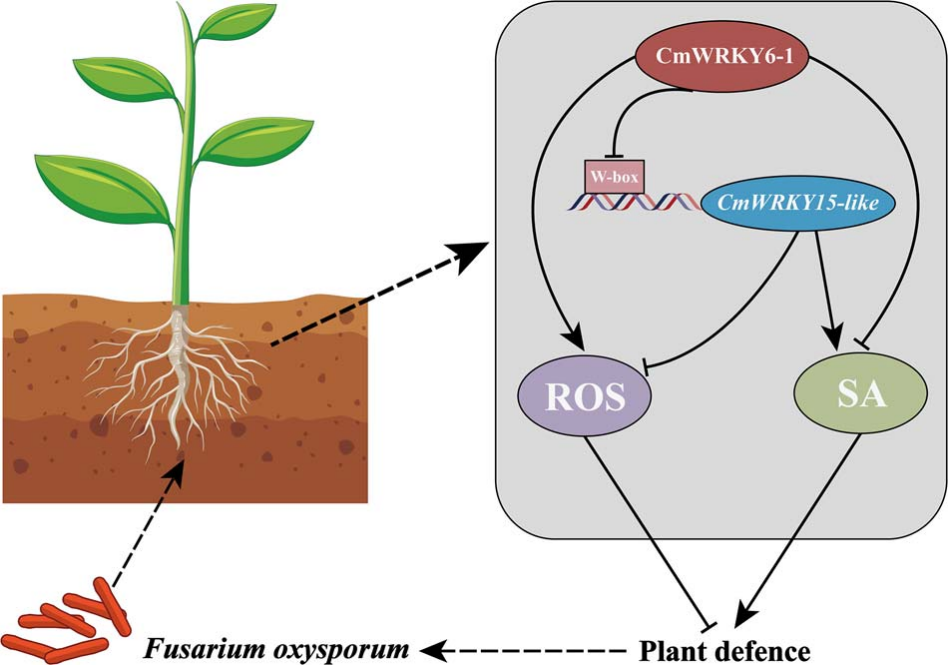
Schematic representation of the mechanism of the CmWRKY6–1–CmWRKY15-like cascade in chrysanthemum in response to *F. oxysporum* infection.

In the present study, we found that *CmWRKY6–1* inhibited the expression of *CmWRKY15-like* and that *CmWRKY6–1* had opposite disease resistance functions as *CmWRKY15-like*. In addition, both *CmWRKY6–1* and *CmWRKY15-like* can regulate ROS and SA pathways. ROS and SA pathways may be involved in the immune regulatory mechanisms of chrysanthemum under biotic or abiotic stresses [28, 33, 50, 51]. Moreover, SA regulates ROS and antioxidant balance [28, 52]. In chrysanthemum, *CmWRKY15–1* positively regulates resistance to white rust by increasing ROS scavenging and SA levels [37, 38]. Therefore, we proposed the hypothesis that CmWRKY6–1–CmWRKY15-like cascades reduces resistance to *F. oxysporum* in chrysanthemum by regulating the ROS and SA pathways (Fig. 7). This is currently under investigation in our lab.

## Materials and methods

### Plant materials

We used the chrysanthemum cultivar ‘Jinba’ and *CmWRKY6–1* transgenic lines provided by the Chrysanthemum Germplasm Resource Preserving Center, Nanjing Agricultural University (Nanjing, China). The plant material was planted in a matrix of nutrient soil mixed with vermiculite (*v*:*v* = 1:2) under an ambient photoperiod of 16/8 h (light/dark), 25°C and 70% humidity.

### Inoculation of *F. oxysporum*

*F. oxysporum* was isolated from the chrysanthemum cultivar, ‘Jinba’ [33]. Fungal cakes were inoculated into potato dextrose agar culture, incubated at 28°C for six days, split into 0.7-cm cakes, inoculated in 500 mL of PDB, and incubated for five days at 28°C and 180 rpm. Next, the chrysanthemum roots were lightly wounded with scissors and immersed in a spore suspension at a concentration of 10^7^ CFU/mL for 30 min. When chrysanthemums were set, they were inoculated with 1 × 10^7^ spores per gram of substrate soil. Finally, the chrysanthemums were cultured in a phototemperature chamber under 16/8 h (light/dark) cycle at 28°C and 80% humidity.

### Isolation and sequence analysis

RNA from ‘Jinba’ was extracted and reverse transcribed into cDNA. ORF sequences of *CmWRKY6–1* and *CmWRKY15-like* were amplified using cDNA as the template and the primers CmWRKY6–1-F/R and CmWRKY15-like-F/R (Table S3, see online supplementary material). We downloaded the homologous sequence of CmWRKY6–1 from GenBank (https://www.ncbi.nlm.nih) and constructed a phylogenetic tree by the neighbor-joining method using MEGA X with 1000 bootstrap replications. We used the DNAMAN software to perform homologous sequence comparisons.

### Subcellular localization

pORE-R4-*CmWRKY6–1* and pORE-R4-*CmWRKY15-like* vectors were constructed. 35S::D53-RFP, pORE-R4-*CmWRKY6–1* and pORE-R4-*CmWRKY15-like* were transfected into *Agrobacterium tumefaciens* strain, GV3101. They were then injected into tobacco leaves via transient transformation and cultured for one day in the dark and two days in the light before observing the fluorescence signal under a confocal microscope.

### Transactivation assays

Construction of pGBKT7-*CmWRKY6–1*. pGBKT7-*CmWRKY6–1*, positive control pCL1, and negative control pGBKT7 were transferred into the Y2H yeast receptor state. The positive control yeast was coated with SD/Leu-deficient media, and the negative control and pGBKT7-*CmWRKY6–1* yeast were coated with SD/Trp-deficient media. After 4–5 d at 28°C, colonies were picked and spotted on SD/Ade-His-deficient media with or without X-α-gal. Yeast growth was observed by capturing photographs after 3–5 d.

Chrysanthemum protoplasts were extracted from the leaves of chrysanthemum histoculture seedlings grown for approximately one month [53]. The luminescence of LUC was measured using a GLOMAX®-20/20 instrument.

### qRT-PCR

Quantitative primers were designed using the Primer Premier software (5.0) (Table S3, see online supplementary material). *CmEF1α* was selected as the reference gene [54, 55]. Each sample was replicated thrice, and the results were calculated using the 2 ^-∆∆ct^ method [56].

### Chrysanthemum transformation and phenotype analysis

pORE-R4-*CmWRKY6–1*, FVuv-R4-*CmWRKY6–1*, and amiR-*CmWRKY15-like* vectors were constructed, transferred into *A. tumefaciens* EHA105, and transformed into chrysanthemum using a previously described method [57, 58]. DNA of the transgenic lines was extracted and identified at the DNA level using vector and gene primers (Table S3, see online supplementary material). RNA from the transgenic lines was extracted and reverse transcribed to cDNA, and the expression level was determined using quantitative primers for the gene (Table S3, see online supplementary material).

‘Jinba’, which had been cut for 40 d, was inoculated with *F. oxysporum*, and the DSI (Table S5, see online supplementary material) was recorded when obvious morbidity phenotype was observed [33].

### RNA-seq analysis

Samples were collected from the pORE-R4-*CmWRKY6–1* and WT roots 0, 3, and 72 h after inoculation with *F. oxysporum*; each sample contained three biological replicates. Transcriptome sequencing was performed using Illumina Novaseq6000 (Gene Denovo Biotechnology Co., Guangzhou, China). Correlation analysis was performed using R. DESeq2 software to identify DEGs between the two groups. DEGs were mapped to GO terms in the GO database (http://www.geneontology.org/) and KEGG in the KEGG database (https://www.kegg.jp/). False-discovery rate (FDR) ≤ 0.05. Transcripts were transformed into FPKM values with RSEM [59]. All analyses were conducted using the omicsmart platform (https://www.omicsmart.com/).

### ROS and SA analysis of chrysanthemum

Roots and basal leaves of plants were collected after infection. Leaves were stained with DAB and NBT. Briefly, the diseased leaves were immersed in DAB solution for 12 h in the dark, decolored with alcohol, and finally photographed and stored. The diseased leaves were soaked in NBT solution for 12 h, decolorized with alcohol, photographed, and stored. Enzymatic activity were measured using the corresponding kits (Comin, Suzhou, China). We also determined the endogenous SA levels in the roots using the SA kit (Lengton Bioscience Co, Shanghai, China).

### Yeast one-hybrid assay

‘Jinba’ DNA was extracted and primers (Table S3, see online supplementary material) were designed to clone the *CmWRKY15-like* promoter sequence. *CmWRKY15-like* promoter sequence was inserted into the pHIS2 vector. pGADT7-*CmWRKY6–1* vector was constructed. pGADT7-*CmWRKY6–1* and pHis2-*CmWRKY15-like*_pro_ vectors were co-transformed into the yeast strain, Y187. pGADT7 and pHis2-*CmWRKY15-like*_pro_ co-transformed yeast were the negative controls. After transformation, the yeast cells were cultured in SD/Trip/His/Leu-deficient medium. After three days, the yeast were picked and transferred to SD/Trip/His/Leu-deficient medium with 90 mM and without 3-amino-1,2,4-triazole, incubated for three days, and photographed for observation.

### EMSA

pGEX-5X-1-*CmWRKY6–1* vector was constructed. Cells were incubated at 16°C. Probes and mutation probe primers (Table S3, see online supplementary material) were designed and the primer pairs were labeled. CmWRKY6–1 binding to *CmWRKY15-like*_pro_ was validated using the EMSA kit [50].

### Luciferase assays

*CmWRKY15-like* promoter was inserted into the pGreenII 0800-Luc vector and transformed into *A. tumefaciens*. pORE-R4-*CmWRKY6–1* and pGreenII 0800-Luc-*CmWRKY15-like*_pro_ were transiently transfected into the tobacco leaves. pORE-R4 empty and pGreenII 0800-Luc-*CmWRKY15-like*_pro_ were the negative controls. Cells were cultured in the dark for 24 h, then in the light for 48 h. Leaves were sprayed with D-fluorescein sodium salt and observed using a Tanon 5200 multi-imaging device (Tanon, Shanghai, China).

pORE-R4-*CmWRKY6–1* and pGreenII 0800-Luc-*CmWRKY15-like*_pro_ were co-transferred into chrysanthemum protoplasts to determine LUC/REN values [50]. pORE-R4 and pGreenII 0800-Luc-*CmWRKY15-like*_pro_ were the negative controls. Each experiment was repeated thrice.

### Statistical analyses

Data analysis were conducted using SPSS. Analysis of variance and *t*-tests were used to analyse all data to determine the significant differences.

## Acknowledgements

This research was supported by National Natural Science Foundation of China (32072603), China Agriculture Research System (CARS-23-A18), The JBGS Project of Seed Industry Revitalization in Jiangsu Province [JBGS(2021)094] and Jiangsu Agriculture Science and Technology Innovation Fund [CX(22)2033].

## Author contributions

S.Z., Y.Liu., Z.G., Y.Liao., S.C., W.F., F.C., and W.M. designed the research. W.M., X.X., Y.W., and Y.Y. performed experiments. W.M. and L.G. analysed data. W.M. wrote the manuscript. All authors read and approved the final manuscript.

## Data availability

All transcriptomic sequencing data associated with this study have been submitted to the NCBI SRA under the accession number PRJNA946259.

## Conflict of interest statement

The authors declare that they have no conflicts of interest.

## Supplementary data

Supplementary data is available at *Horticulture Research* online.

## Supplementary Material

Web_Material_uhad101
