# Supplementary material for: CmWRKY6–1–CmWRKY15-like transcriptional cascade negatively regulates the resistance to fusarium oxysporum infection in Chrysanthemum morifolium
Source: Hortic Res. 2023 May 10;10(7):uhad101. doi: 10.1093/hr/uhad101 (PMC10419886; doi:10.1093/hr/uhad101)
Supplement: Web_Material_uhad101 [file web_material_uhad101.zip › Supplementary Figures .docx]

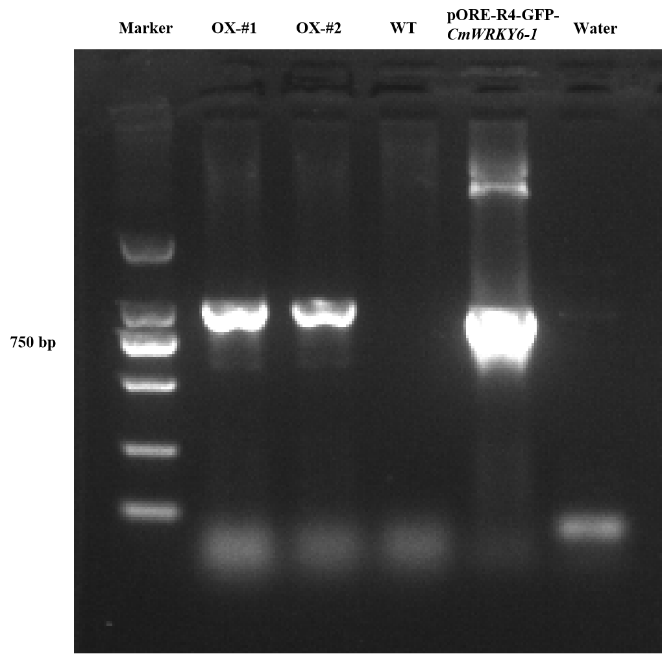


**Figure S1.** Identification of *CmWRKY6-1* overexpression lines at the DNA level.


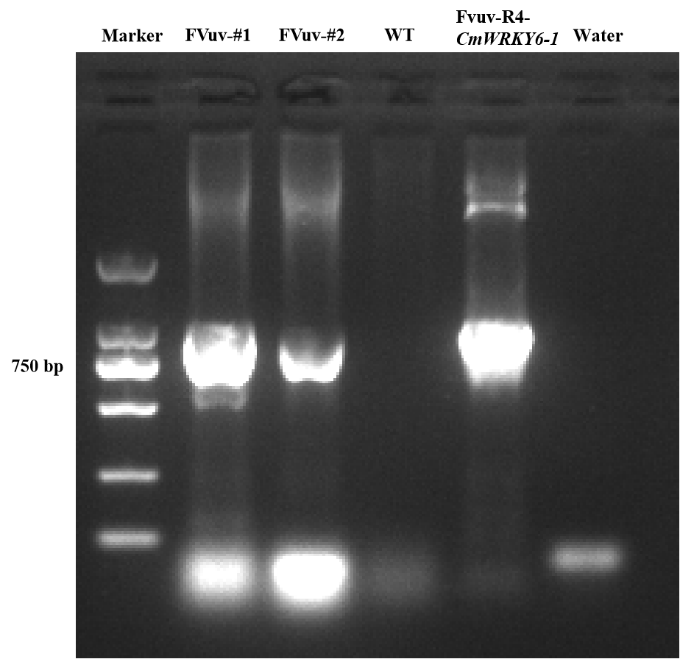


**Figure S2.** Identification of *CmWRKY6-1* interfering lines at the DNA level.


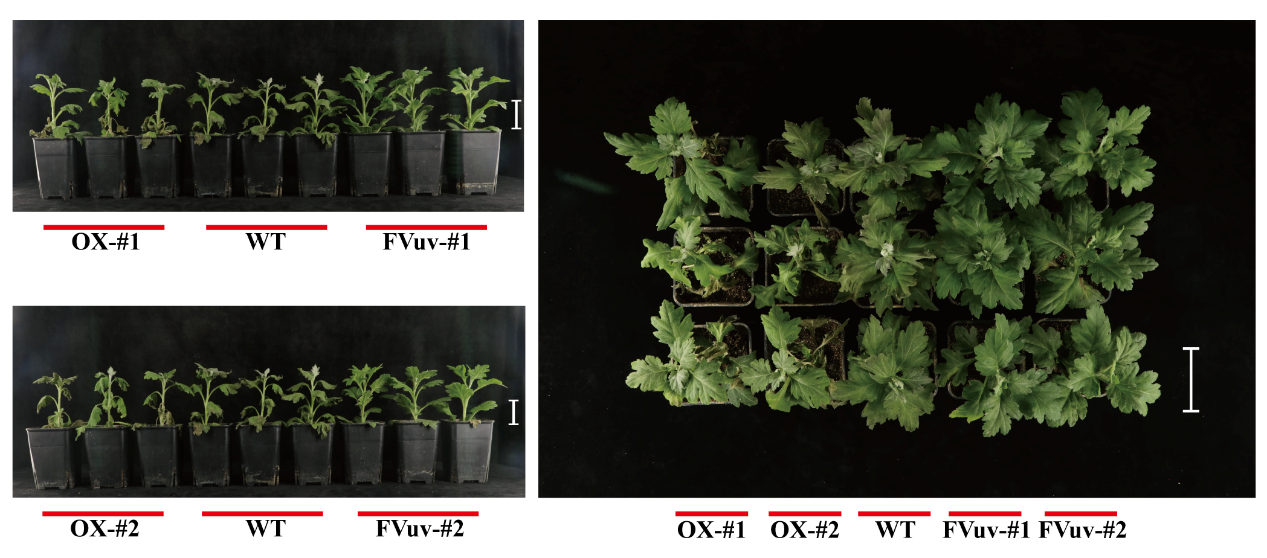


**Figure S3.** Phenotypic observations of the *CmWRKY6-1* transgenic lines and WT after inoculation with *F. oxysporum*. Bars = 5 cm.


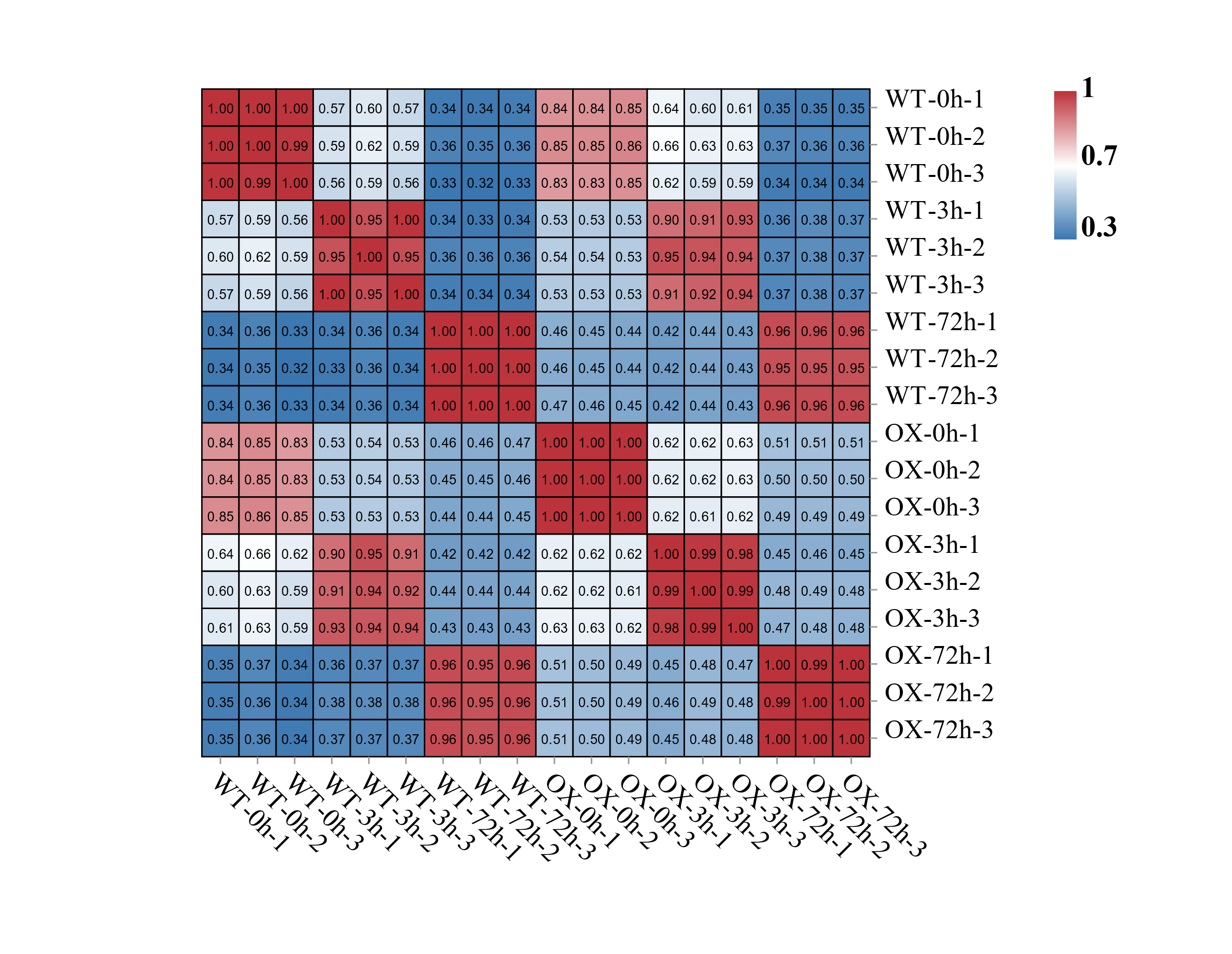


**Figure S4.** Correlation coefficients among transcriptome samples.


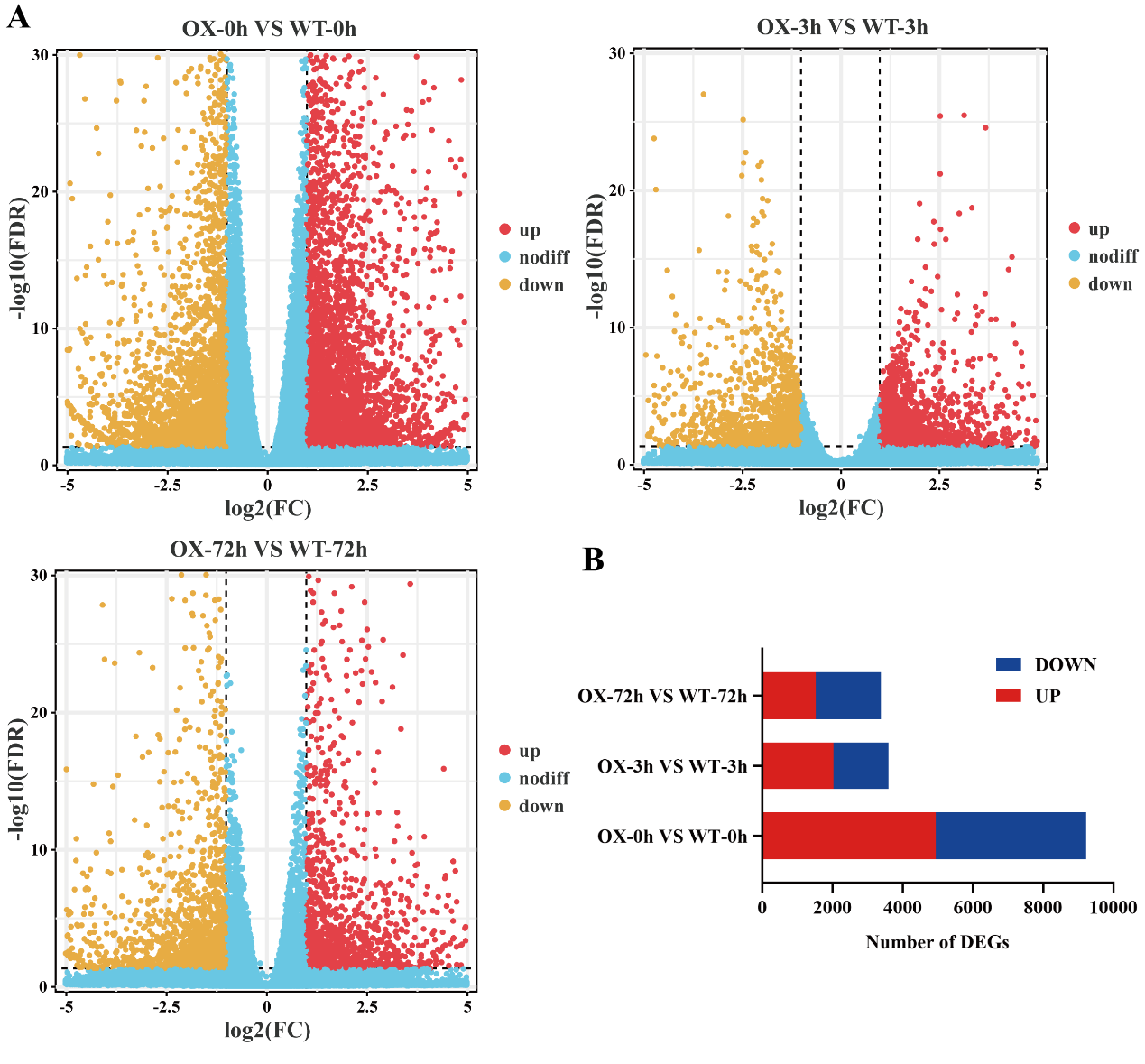


**Figure S5.** Transcriptome analysis of differentially expressed genes (DEGs). **A** Volcano plot of DEGs between samples. **B** Numbers of DEGs up- and downregulated in the samples.

**
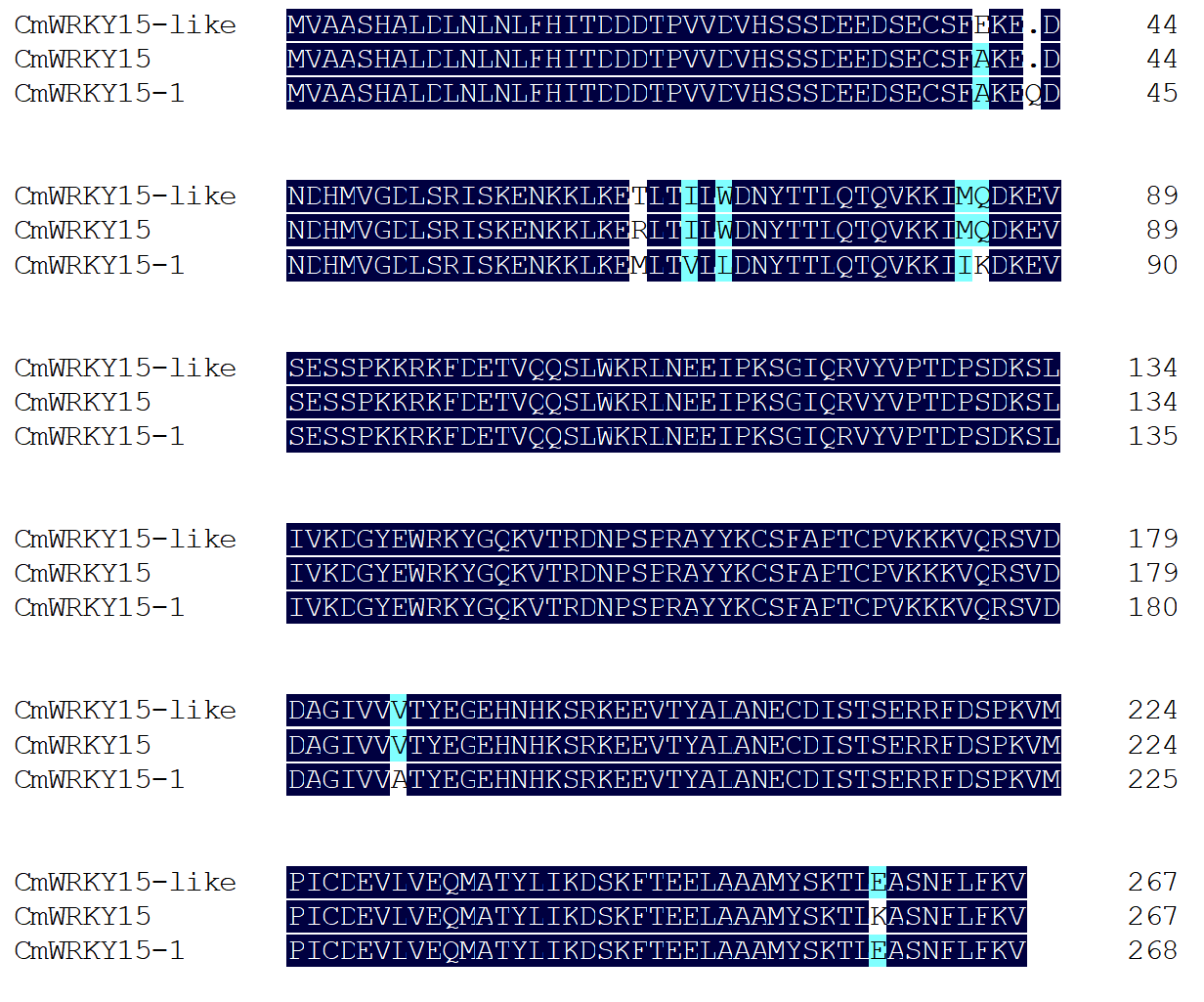
Figure S6.** Sequence alignment of CmWRKY15-like, CmWRKY15, and CmWRKY15-1.

**Figure S7.**
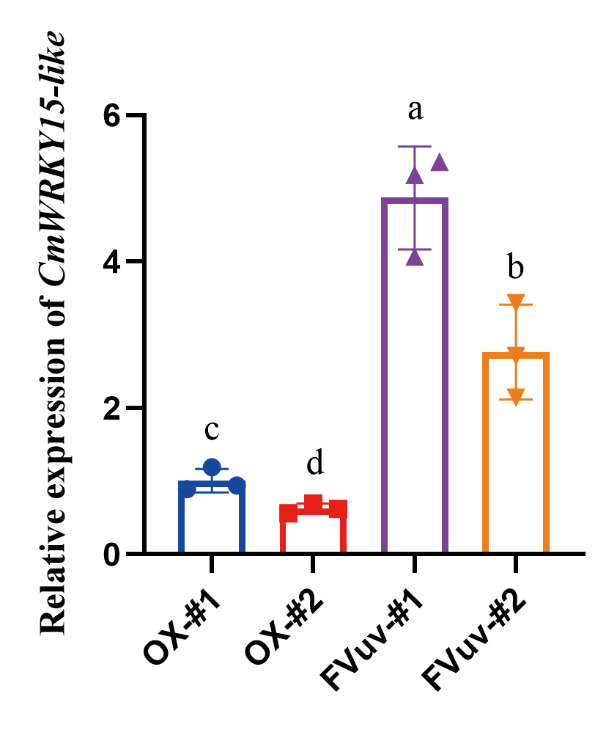
 Relative expression of *CmWRKY15-like* in *CmWRKY6-1* transgenic lines, primers: *CmWRKY15-like*-qRT-PCR-F, *CmWRKY15-like*-qRT-PCR-R (Supplementary Data Table S3).


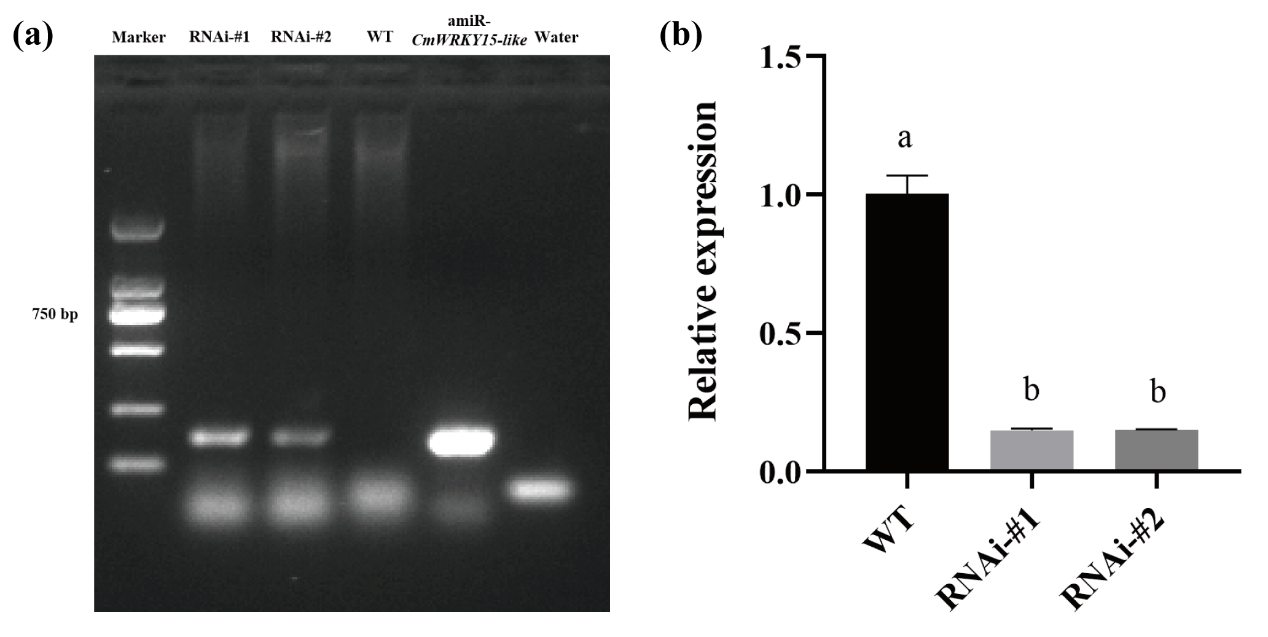


**Figure S8.** Identification of *CmWRKY15-like* transgenic lines. **A** Identification of *CmWRKY15-like* transgenic lines at the DNA level, primers: amiR-F, amiR-R (Supplementary Data Table S3). **B** Identification of *CmWRKY15-like* transgenic lines at the RNA level.
