# Supplementary material for: CmWRKY6–1–CmWRKY15-like transcriptional cascade negatively regulates the resistance to fusarium oxysporum infection in Chrysanthemum morifolium
Source: Hortic Res. 2023 May 10;10(7):uhad101. doi: 10.1093/hr/uhad101 (PMC10419886; doi:10.1093/hr/uhad101)
Supplement: Web_Material_uhad101 [file web_material_uhad101.zip › Table S2.docx]

| **Sample** | **Raw reads** | **Clean reads** | **Q20 (%)** | **Q30 (%)** | **GC (%)** |
| --- | --- | --- | --- | --- | --- |
| WT-0h-1 | 49,781,718 | 49,482,914 | 96.50% | 90.85% | 42.90% |
| WT-0h-2 | 49,678,834 | 49,416,822 | 97.24% | 92.33% | 42.86% |
| WT-0h-3 | 49,953,480 | 49,646,880 | 96.69% | 91.23% | 42.76% |
| WT-3h-1 | 40,956,290 | 40,755,392 | 97.90% | 93.71% | 43.72% |
| WT-3h-2 | 40,728,722 | 40,522,292 | 98.17% | 94.49% | 45.05% |
| WT-3h-3 | 35,401,912 | 35,246,216 | 97.98% | 93.92% | 43.61% |
| WT-72h-1 | 59,810,426 | 59,406,370 | 97.17% | 92.34% | 45.95% |
| WT-72h-2 | 59,983,222 | 59,587,004 | 97.38% | 92.83% | 46.29% |
| WT-72h-3 | 59,713,338 | 59,328,212 | 97.14% | 92.24% | 45.87% |
| OX-0h-1 | 59,706,418 | 59,393,184 | 97.36% | 92.65% | 43.05% |
| OX-0h-2 | 59,862,620 | 59,500,618 | 96.76% | 91.39% | 42.99% |
| OX-0h-3 | 59,636,264 | 59,268,264 | 96.64% | 91.13% | 42.96% |
| OX-3h-1 | 59,633,794 | 59,251,598 | 97.51% | 93.08% | 46.19% |
| OX-3h-2 | 59,761,176 | 59,397,154 | 97.24% | 92.49% | 45.50% |
| OX-3h-3 | 59,888,336 | 59,513,326 | 97.25% | 92.44% | 45.06% |
| OX-72h-1 | 59,974,842 | 59,644,242 | 97.22% | 92.42% | 46.63% |
| OX-72h-2 | 48,877,326 | 48,656,042 | 97.96% | 93.97% | 46.84% |
| OX-72h-3 | 44,674,328 | 44,414,758 | 97.68% | 93.46% | 46.54% |

**Table S2.** RNA-Seq data and corresponding quality control
