# Supplementary material for: CmWRKY6–1–CmWRKY15-like transcriptional cascade negatively regulates the resistance to fusarium oxysporum infection in Chrysanthemum morifolium
Source: Hortic Res. 2023 May 10;10(7):uhad101. doi: 10.1093/hr/uhad101 (PMC10419886; doi:10.1093/hr/uhad101)
Supplement: Web_Material_uhad101 [file web_material_uhad101.zip › Table S3.docx]

**Table S3.** Primer sequences

| **Primer** | **Sequence** |
| --- | --- |
| *CmWRKY6-1*-F | ATGAATGCTCTTCCGTTGTC |
| *CmWRKY6-1*-R | TCATGTGTTTGCAGACTGAG |
| *CmWRKY15-like*-F | ATGGTGGCTGCATCACATGC |
| *CmWRKY15-like*-R | TTAACATACTTTGAATAAAA |
| *CmWRKY6-1-*qRT-PCR-F | TTGGGTCGACAAGGTCAACT |
| *CmWRKY6-1-*qRT-PCR -R | CCTGTGCTTCCTCTTCTTGC |
| *CmWRKY15-like-*qRT-PCR -F | TGGCTGCATCACATGCCTTAGA |
| *CmWRKY15-like-*qRT-PCR -R | TCGCTGTCCTCCTCATCAGAACT |
| *CmEF1α-*F | TTTTGGTATCTGGTCCTGGAG |
| *CmEF1α-*R | CCATTCAAGCGACAGACTCA |
| 35S | GACGCACAATCCCACTATCC |
| amiR-F | GACTGGGGATTTAAGTCGGATAATCAAAGAGAATCAATGA |
| amiR-R | GACTAGGGATTTAAGACGGATATTCACAGGTCGTGATATG |
| *CmWRKY15-like*_pro_*-*F | AAGGGATCAATTTACATCCTCCATT |
| *CmWRKY15-like*_pro_*-*R | CTATGTATTTCTACACACACAAAGA |
| EMSA-probe-F | TCATAAGTTATATTCAAATATTGACAAATTTTGATACTTTGAACC |
| EMSA-probe-R | GGTTCAAAGTATCAAAATTTGTCAATATTTGAATATAACTTATGA |
| EMSA-mprobe-F | TCATAAGTTATATTCAAATAAAAAAAAATTTTGATACTTTGAACC |
| EMSA-mprobe-R | GGTTCAAAGTATCAAAATTTTTTTTTATTTGAATATAACTTATGA |
| evm.TU.scaffold_72.66-qRT-PCR-F | AGAAGTCGAGGCCGTAAGAG |
| evm.TU.scaffold_72.66- qRT-PCR-R | ACCGGGTGAAGTCACATTCT |
| evm.TU.scaffold_7835.98-qRT-PCR-F | CTTGGTGGCACTCTGTCAAG |
| evm.TU.scaffold_7835.98-qRT-PCR-R | TGGGTGAAGCTCACCGTTAT |
| evm.TU.scaffold_1650.57-qRT-PCR-F | TGAGCTTCACCCATCTCGTT |
| evm.TU.scaffold_1650.57-qRT-PCR-R | GGTAAGTTGCGCTACAAGGG |
| evm.TU.scaffold_1703.36-qRT-PCR-F | CACCGTCACAAGGTGCATTT |
| evm.TU.scaffold_1703.36-qRT-PCR-R | AATGTCGCATAGGCAACTGA |
| evm.TU.scaffold_11771.58-qRT-PCR-F | CAGTTGCCTATGCGACGTTT |
| evm.TU.scaffold_11771.58-qRT-PCR-R | TGGCCATCCCGTTTCTGATA |
| evm.TU.scaffold_8788.18-qRT-PCR-F | CACCGTCACAAGGTGCATTT |
| evm.TU.scaffold_8788.18-qRT-PCR-R | AATGTCGCATAGGCAACTGA |
| evm.TU.scaffold_3677.49-qRT-PCR-F | TACACTGTTTGGCCCGGTAT |
| evm.TU.scaffold_3677.49-qRT-PCR-R | CCGGATGGAGCTTGTAAGGA |
| evm.TU.scaffold_670.241-qRT-PCR-F | GTCCAGCATCAACAAGCCAA |
| evm.TU.scaffold_670.241-qRT-PCR-R | GAATGGGTGCTCGTGGAATC |
| evm.TU.scaffold_1289.286-qRT-PCR-F | CACAGGTGGTACCGGACATA |
| evm.TU.scaffold_1289.286-qRT-PCR-R | CGCCGCTACAACAATACTCC |
| evm.TU.scaffold_1621.173-qRT-PCR-F | CTAGCGACGGAGATGGATCA |
| evm.TU.scaffold_1621.173-qRT-PCR-R | ACTGCATGTTTGAGGTGTCG |
