# Supplementary material for: CmWRKY6–1–CmWRKY15-like transcriptional cascade negatively regulates the resistance to fusarium oxysporum infection in Chrysanthemum morifolium
Source: Hortic Res. 2023 May 10;10(7):uhad101. doi: 10.1093/hr/uhad101 (PMC10419886; doi:10.1093/hr/uhad101)
Supplement: Web_Material_uhad101 [file web_material_uhad101.zip › Table S4.docx]

| **Gene** | **Sequence** |
| --- | --- |
| *CmWRKY15-1*_pro_ | AAGGGATCAATTTACATCCTCCATTTTTATTAGATCTAATGGATGAGATTAATCCCAACAATTAAAAAAACTCACTTTCATTCTTTTTTTTTTTTCAAATAGAAATATCTTTCAAACCGTTAATCGTCAAGCGAAAAAAAATATACCATTACGACGGGCATTTAGTTATCTTTCTATAGATAGGCACTTTGATATACTTTCGACAATTTTTTTTTTCGTGTTTTTCACGTTACTGAAATTTGCATGAAAAAATCATTTATGATTTAACACTAATTTGAATACCACATGTGATATTAATGTTAAATCATATATGATATGTATATCAAATGTGATTTATCATATATGATTTAACACTAGTATCACATATGATATTACATATCATATGTGATTTAACACTAATGTCATATGTGATTTTCAAATTAATGTTAAATCATATATGATATTTAATGCAAATTCCAGTTTCGAAGAAAAATGAAAAAAAGACGAAAAAACGATTTTTTTCTTTTTTCAAAAGTATATCAAAGTGCCCGTCTATAGAAAGATAATTAAATGCTCGTCGCGATGGTGTAGTTATTTTTGCTTAACGATTAACGGTTTAAGAGATATTTAAATTATAAAAAATATGTGAAATAATAAAATGCTTTTAATCTCAGTCATTGATTTATTTTTAGATGGATGGTGGAGATGAGTCCCTTGATATGTAATAAAAGTTTGGTATGTAAAATAACTCTCCTCTAAAATAAATATCTATTTTGTGACCAAGAGCCATATAGTTTATCTTTCTTACGGCGTAACATCATAACTCTCGTTATGCTGTAGAGCTCTCGAATAAGGATTTATTTAGACGTGTTAATTGTTACAATGTAAACATGAATATCTTGTTACGACATAGAGGCTTCGAAAGCTGATTTTTTTTTTTTTTTTTTTTTTTATTTATGTTTAAGTCAGTTTTTTACTATAAGGTTAATGTTAGCGAAGATAATGAAATCCTATTTAAAAAAAATAACGAGAGTCCTCAAGATTGTTATGTTGATGGAGATAATCACAATATTTTTTAATGTAAAAGTTTGGGATGGTTTAGAAACAAATATTAAAGAGCTACTTGTAAGGAGGTTTTTAGAGAAACTAATATAAATTATGTAAAAGATAAATTTAGTAACATTTTATTTAGTGAATAAAAATCGTCGTAGAGACGAGTTGCCTAGCTTGAACATGTTCCAAAACCTTAAAAAAATATATATTGTTTGTTTACAAGGGGCATAATTCACTCGGCTCGTCCAAGACATTCGAAATCTCAGAACCGGCCCTGATAACAATCTAAGCATCTGATCTTAAACTATGAAGCAAAAGTTTGGTTTCATACGTATAAAGGTTAGCAATCAAAAATAGGAAGAGGTCACCAAGCTCTAAATTTGATGTTATTTTACATTAATTTTCTTGTGTAAAGTGTATATTTGATTTACAAAACTTAAATTATAATAAAAATTTGAATTAATATCATAAAATAGCAACATATTTTGCAAAAACTTTAATTTTGATAACTAAACTTCTTTTTGTTCCACTTTGGTTATTATACTTATTGAGGTGATTCATAATAATTATTATCTTTTTTGAATTTTAATCCGGTAAACTGATATAACCGCTCAAAATATATAACATATCATAAGTTATATTCAAATATTGACAAATTTTGATACTTTGAACCGATTTATTAAAGAAACAAGGTAAAAATAAGAAAGTACAATAAGTGGAAATTCTTCTCTTCTTCATAACACATCATCAAAAATACTTCAAAATTTTGTATAACTTACGTTATTTTAGAGATTTTGACGAGTTTTACCGATCATTCATAAAAAGTCGAGAAAATAGTAGCCATTATGACTTACACATGTACGTATGATCACTAAAATGAATAAAAATAATAATAATTACCAATATAAACAAACGTTCCTATTTTGTAACATTAATCCTAGAAATTTATAAAAATCCCAAAGACTTTTTGCAAACCTTGGTAACGTGTCTCTCTTTGTGTGTGTAGAAATACATAG |

**Table S4.** *CmWRKY15-like* promoter sequence
