# Supplementary material for: CmWRKY6–1–CmWRKY15-like transcriptional cascade negatively regulates the resistance to fusarium oxysporum infection in Chrysanthemum morifolium
Source: Hortic Res. 2023 May 10;10(7):uhad101. doi: 10.1093/hr/uhad101 (PMC10419886; doi:10.1093/hr/uhad101)
Supplement: Web_Material_uhad101 [file web_material_uhad101.zip › Table S5.docx]

| **The classification of disease severity** | **Symptom description** | |
| --- | --- | --- |
| 0 | | No disease |
| 1 | | Only one leaf at the base turns yellow or curls |
| 2 | | 1/3-1/2 of the whole plant leaves become yellow and curled or wilted, the plant is slightly shorter |
| 3 | | 1/2-3/4 leaves of the whole plant become yellow and curled or wilted, leaves fall off |
| 4 | | Whole plant leaves gum yellow and curled or wilted, or the plant died |

**Table S5.** Chrysanthemum Fusarium wilt disease incidence grade index
