# Supplementary figures and images for: CmWRKY6–1–CmWRKY15-like transcriptional cascade negatively regulates the resistance to fusarium oxysporum infection in Chrysanthemum morifolium
Source: Hortic Res. 2023 May 10;10(7):uhad101. doi: 10.1093/hr/uhad101 (PMC10419886; doi:10.1093/hr/uhad101)

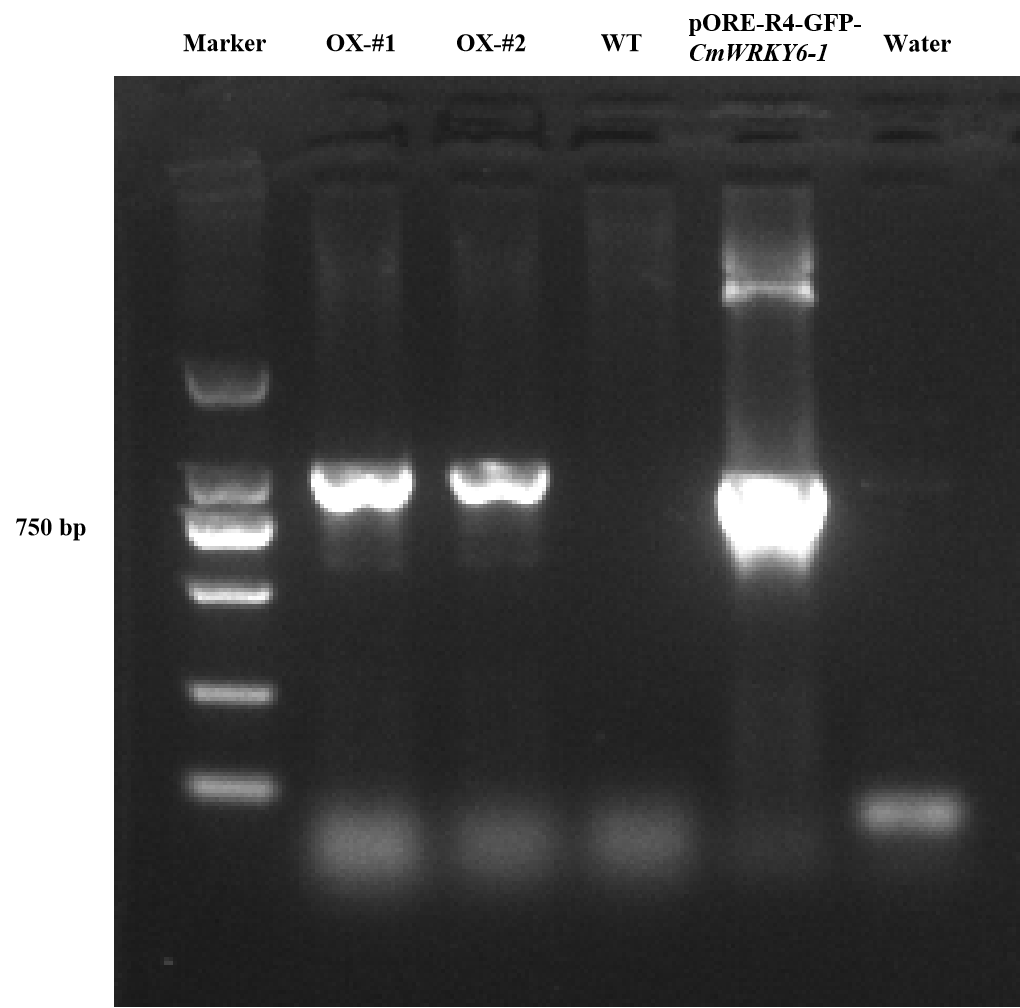

Supplement: Web_Material_uhad101 [file web_material_uhad101.zip › Figure S1.tif]

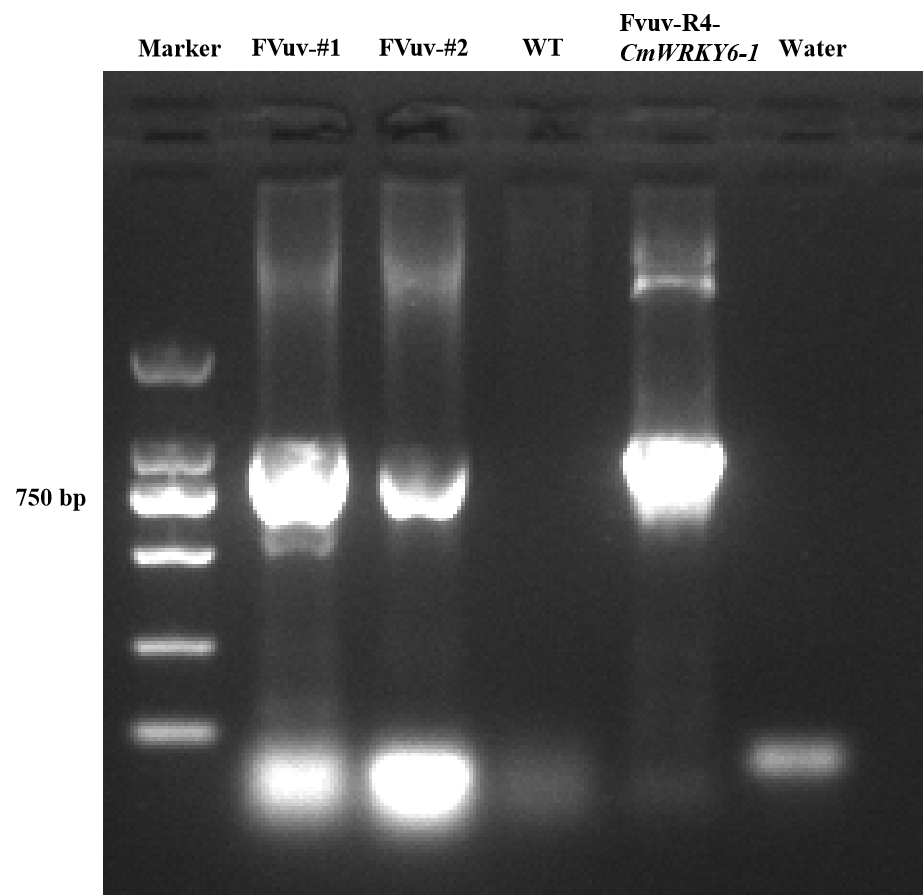

Supplement: Web_Material_uhad101 [file web_material_uhad101.zip › Figure S2.tif]

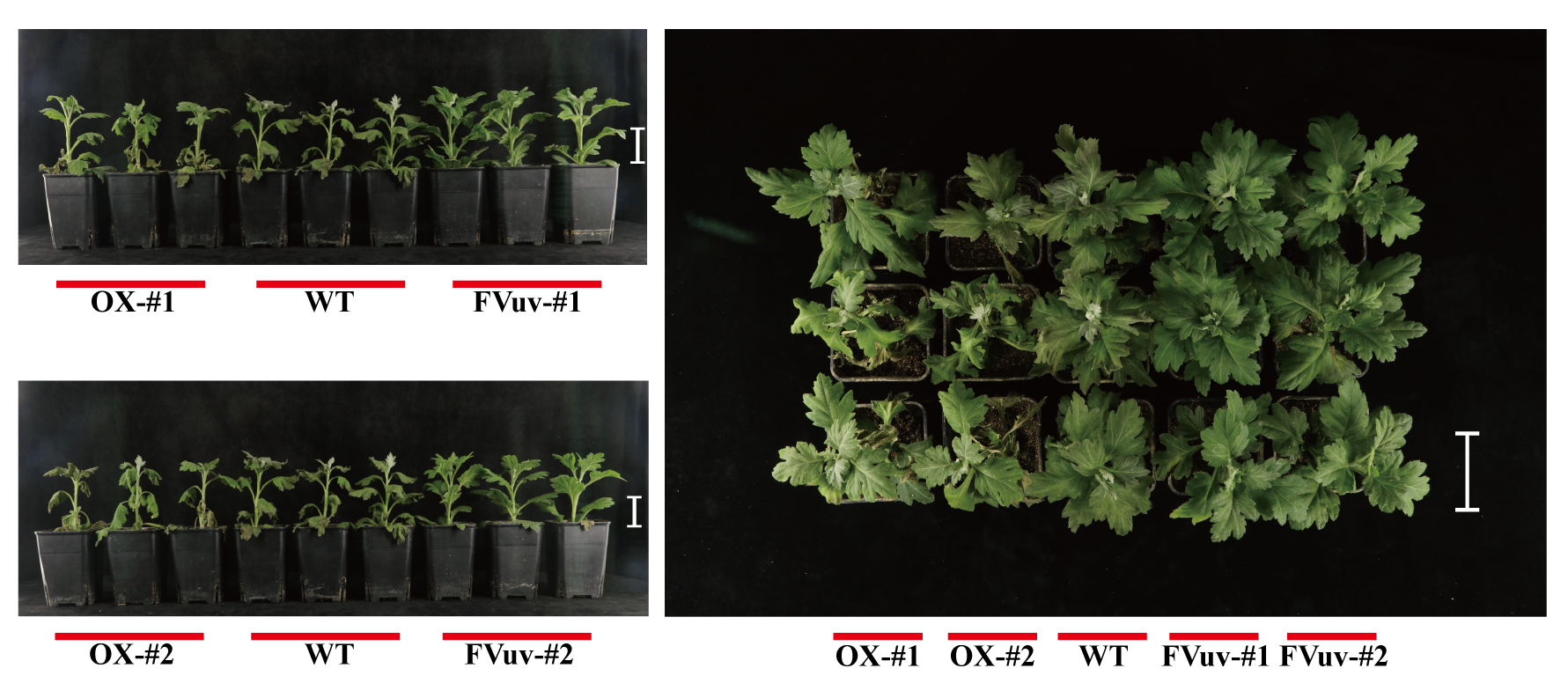

Supplement: Web_Material_uhad101 [file web_material_uhad101.zip › Figure S3.tif]

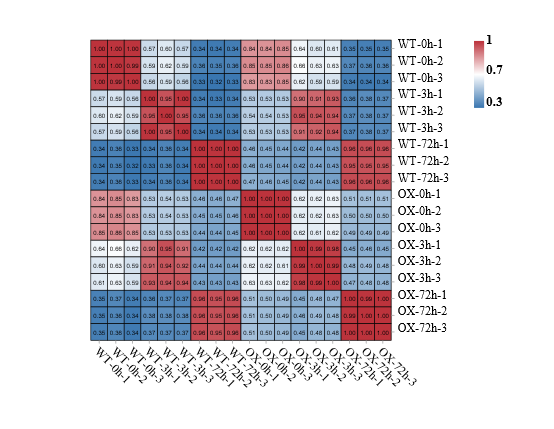

Supplement: Web_Material_uhad101 [file web_material_uhad101.zip › Figure S4.tif]

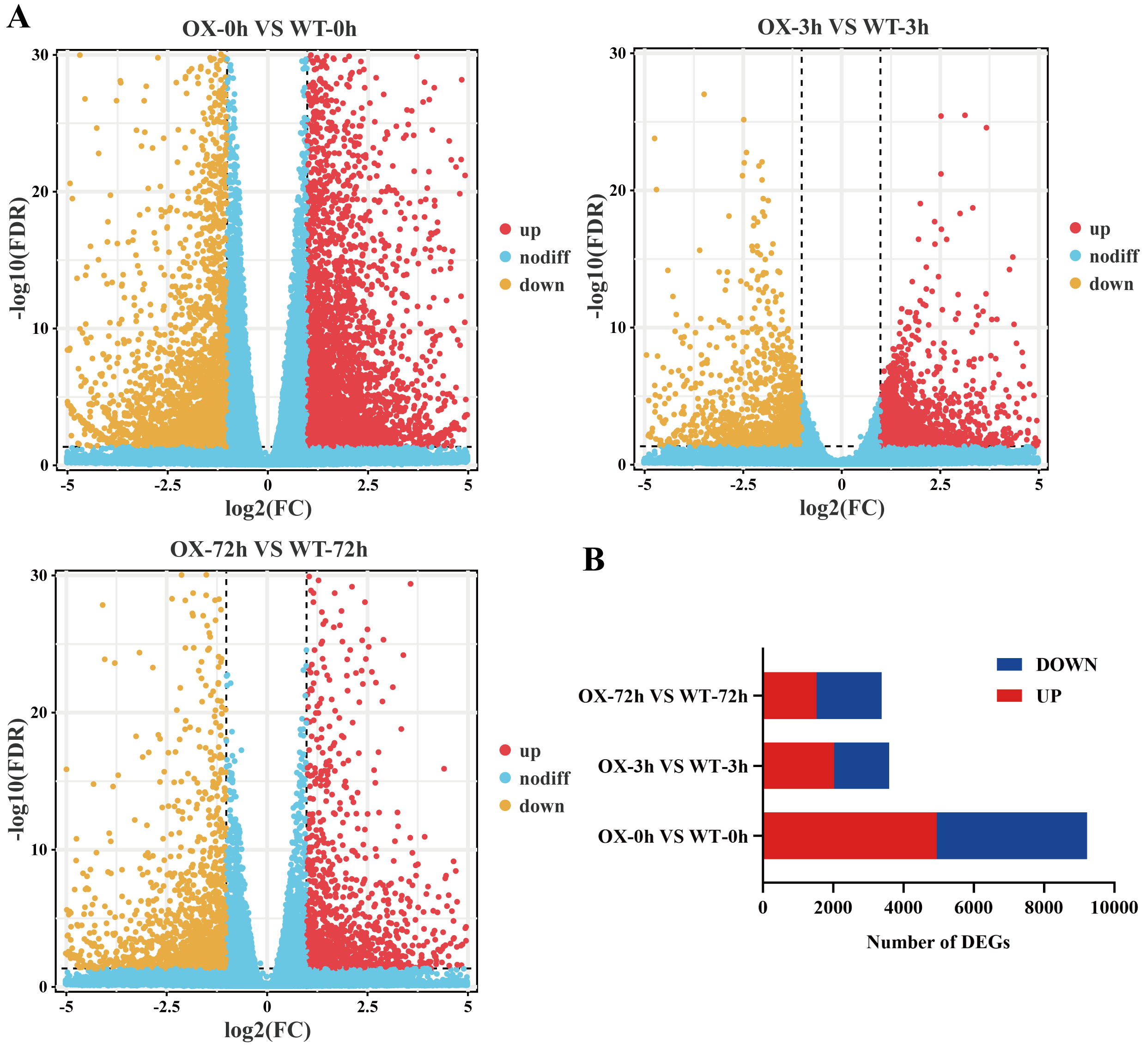

Supplement: Web_Material_uhad101 [file web_material_uhad101.zip › Figure S5.tif]

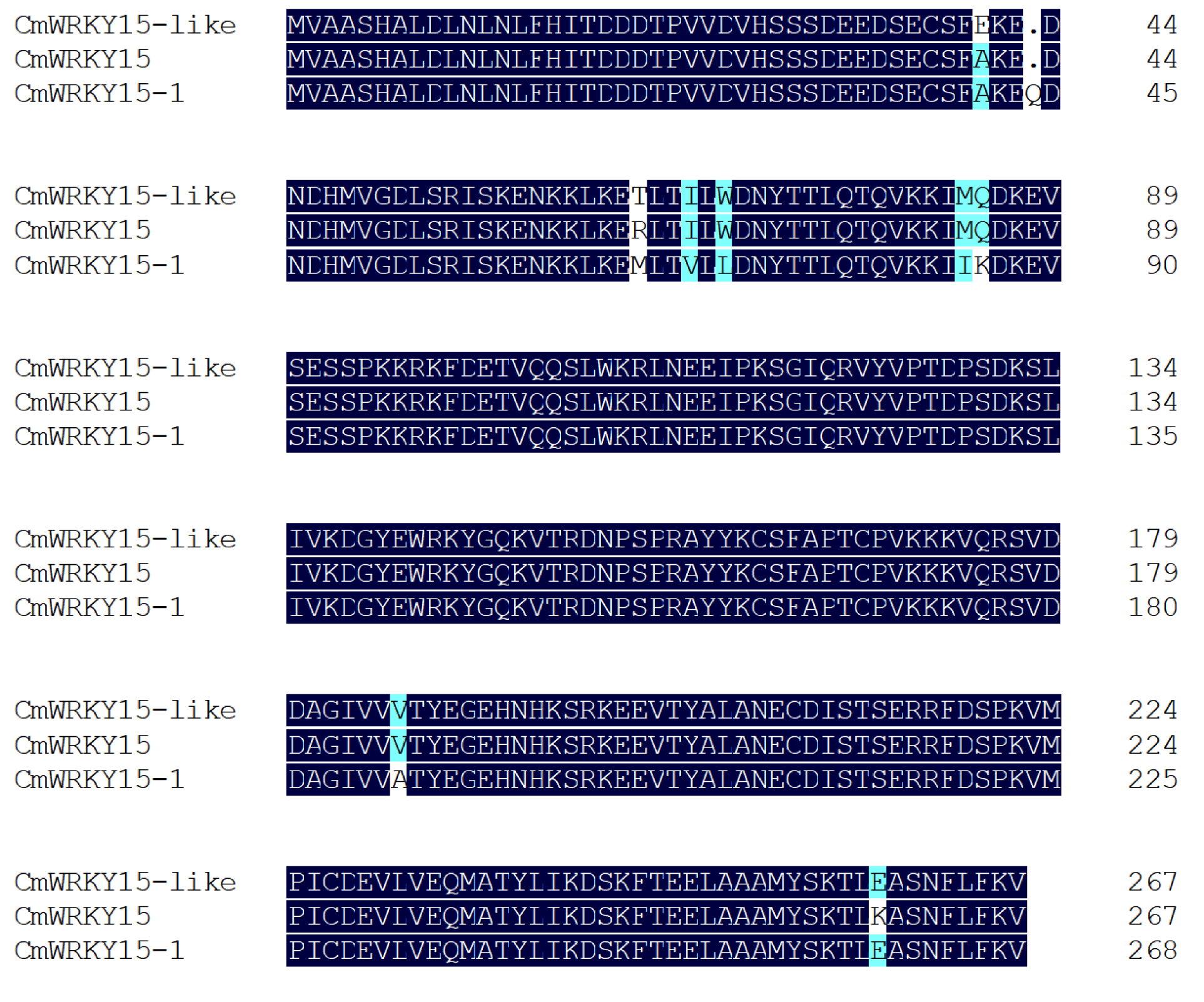

Supplement: Web_Material_uhad101 [file web_material_uhad101.zip › Figure S6.tif]

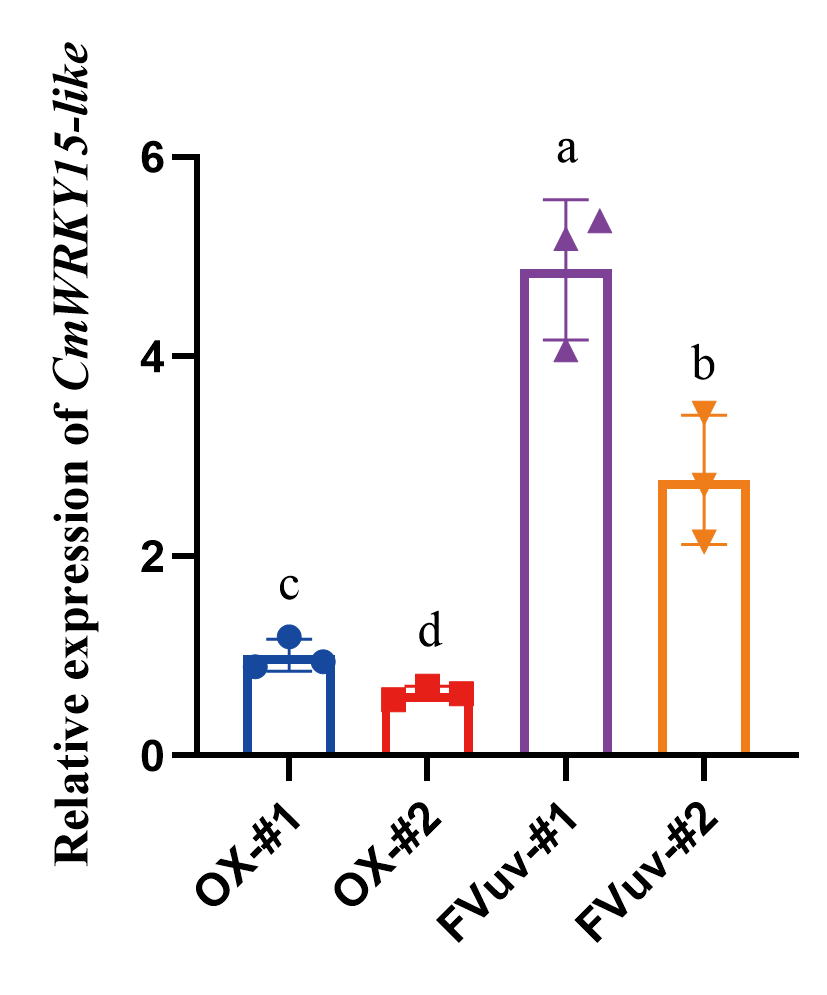

Supplement: Web_Material_uhad101 [file web_material_uhad101.zip › Figure S7.tif]

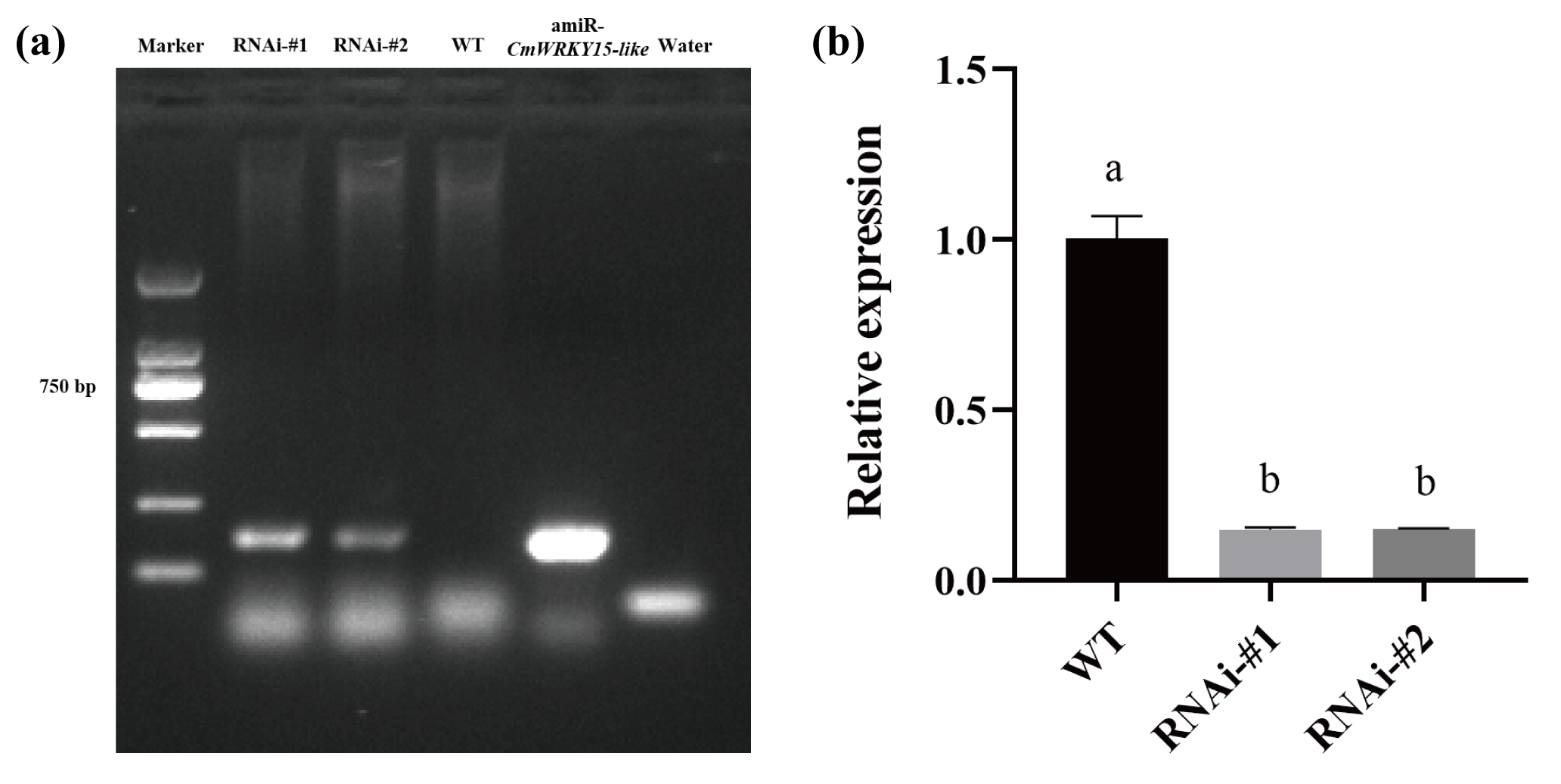

Supplement: Web_Material_uhad101 [file web_material_uhad101.zip › Figure S8.tif]
